# Heterogeneity in schistosomiasis transmission dynamics

**DOI:** 10.1016/j.jtbi.2017.08.015

**Published:** 2017-11-07

**Authors:** Lorenzo Mari, Manuela Ciddio, Renato Casagrandi, Javier Perez-Saez, Enrico Bertuzzo, Andrea Rinaldo, Susanne H. Sokolow, Giulio A. De Leo, Marino Gatto

**Affiliations:** a Dipartimento di Elettronica, Informazione e Bioingegneria, Politecnico di Milano, 20133 Milano, Italy; b Laboratory of Ecohydrology, Ecole Polytechnique Fédérale de Lausanne, 1015 Lausanne, Switzerland; c Dipartimento di Scienze Ambientali, Informatica e Statistica, Università Ca’ Foscari Venezia, 30170 Venezia Mestre, Italy; d Dipartimento ICEA, Università di Padova, 35131 Padova, Italy; e Hopkins Marine Station, Stanford University, Pacific Grove, CA 93950, USA; f Marine Science Institute, University of California, Santa Barbara, CA 93106, USA

**Keywords:** Heterogeneous transmission, Schistosomiasis, Macroparasitic model, Disease control

## Abstract

Simple models of disease propagation often disregard the effects of transmission heterogeneity on the ecological and epidemiological dynamics associated with host-parasite interactions. However, for some diseases like schistosomiasis, a widespread parasitic infection caused by *Schistosoma* worms, accounting for heterogeneity is crucial to both characterize long-term dynamics and evaluate opportunities for disease control. Elaborating on the classic Macdonald model for macroparasite transmission, we analyze families of models including explicit descriptions of heterogeneity related to differential transmission risk within a community, water contact patterns, the distribution of the snail host population, human mobility, and the seasonal fluctuations of the environment. Through simple numerical examples, we show that heterogeneous multigroup communities may be more prone to schistosomiasis than homogeneous ones, that the availability of multiple water sources can hinder parasite transmission, and that both spatial and temporal heterogeneities may have nontrivial implications for disease endemicity. Finally, we discuss the implications of heterogeneity for disease control. Although focused on schistosomiasis, results from this study may apply as well to other parasitic infections with complex transmission cycles, such as cysticercosis, dracunculiasis and fasciolosis.

## Highlights

• Transmission dynamics of schistosomiasis presents multiple heterogeneity sources.
• A comprehensive framework for heterogeneous disease transmission is proposed.
• Heterogeneous multigroup communities can be more prone to parasite transmission.
• Presence of multiple water sources can hinder parasite transmission.
• Spatial and temporal heterogeneities can have nontrivial implications for endemicity.

## 1 Introduction

Transmission heterogeneity is a central issue in the study of infectious disease dynamics; in fact, it has been receiving considerable attention for more than thirty years, with research focusing on the role played by heterogeneity in both short-term epidemic dynamics and long-term transmission maintenance, as well as on the challenges and opportunities that heterogeneity poses to disease control (see e.g. Andreasen, Christiansen, 1989, Bolzoni, Real, De Leo, 2007, Diekmann, Heesterbeek, Metz, 1990, Dushoff, Levin, 1994, Heffernan, Smith, Wahl, 2005, Hethcote, Van Ark, 1987, Lloyd-Smith, Schreiber, Kopp, Getz, 2005, Nold, 1980, van den Driessche, Watmough, 2002, Woolhouse, Dye, Etard, Smith, Charlwood, Garnett, Hagan, Hii, Ndhlovu, Quinnell, Watts, Chandiwana, Anderson, 1997; see also VanderWaal and Ezenwa, 2016 for a recent review). A full understanding of the drivers and the consequences of heterogeneity still represents a major challenge for epidemiology, especially for diseases characterized by indirect transmission, such as vector-borne, water-related, or macroparasitic infections (Hollingsworth et al., 2015).

One such disease for which there is a unanimous scientific consensus about the relevance of heterogeneity on transmission is schistosomiasis, one of the most common parasitic diseases (second only to malaria) and the deadliest among the neglected tropical diseases (Centers for Disease Control and Prevention, 2011). Schistosomiasis is caused by trematode parasites belonging to the genus *Schistosoma*, which need some species of freshwater snails as obligate intermediate hosts. It affects more than 250 million people in tropical and subtropical regions of the developing world, especially in sub-Saharan Africa, which is home to approximately 90% of worldwide cases (World Health Organization, 2017). Human infection occurs through contact with water contaminated with freely swimming, short-lived schistosome larvae, known as cercariae, which are shed by infected snails. Specifically, cercariae can infect exposed humans through skin penetration. Within human hosts, they develop into sexually mature schistosomes. Depending on their species, the resting place of adult parasites is in the blood vessels around the bladder or the intestine of the infected human host. There, schistosomes mate and produce eggs that are excreted through urine or faeces. After reaching freshwater, eggs hatch into so-called miracidia, a second short-lived larval form of the parasite that can infect species-specific snail hosts and undergo asexual replication therein. Infected snails complete the parasite’s life cycle by shedding cercariae into water (see e.g. Colley et al., 2014).

The transmission of schistosomiasis is thus controlled by contact with environmental freshwater infested with parasite larvae. Unsurprisingly, then, the disease is especially prevalent in communities that lack access to piped drinking water and adequate sanitation, and that have to resort to unsafe water sources for their primary needs (Rollinson et al., 2013). Conversely, the availability of adequate water provisioning and sanitation infrastructures may offer protection against schistosomiasis (Grimes et al., 2014). At a regional scale, different communities may be endowed with differential infection risk linked to their geographical and/or socioeconomic context. However, safe water supplies cannot completely avert human contact with environmental freshwater, nor can the presence of adequate sanitation guarantee *per se* its use (Grimes et al., 2015). Agricultural, domestic, occupational and recreational tasks may foster contact with potentially infested water, and thus represent risk factors even in places where water provisioning and sanitation are adequate. Also, differences in behavior and lifestyle represent major potential determinants of transmission heterogeneity (Funk et al., 2015). As a result, the risk of schistosomiasis infection is usually not equally spread even at a local level, i.e. within a community, actually being higher for people whose routinary activities bring them in contact with water, such as fishermen, farmers and women. Overall, however, infection risk is highest among school-aged children, mostly because they are likely to spend a considerable amount of time in unsafe water; also, their immune system may not be fully developed yet (World Health Organization, 2017). Several risk groups may thus be identified within a local community, possibly based on age, sex and/or occupation (e.g. Spear et al., 2004a).

In communities with access to several water sources, even individuals within the same risk group may be endowed with differential infection risk because of their specific water-contact patterns. Different water bodies may represent habitats of different quality for the intermediate snail hosts of the schistosomes, and can thus be relatively heterogeneous in terms of the local snail population abundance they can host (e.g. Gurarie and King, 2005). As such, a comprehensive description of heterogeneity in schistosomiasis transmission also requires an account of individual preferences related to water contacts (e.g. Scott et al., 2003), as well as an ecological assessment of snail density (and possibly infection prevalence) at each of the available water sources (Perez-Saez, Mande, Ceperley, Bertuzzo, Mari, Gatto, Rinaldo, 2016, Gurarie, King, Yoon, Alsallaq, Wang, 2017). Additionally, infection risk may also be heterogeneously distributed over time. Temporal variability of schistosomiasis transmission can arise from the seasonal fluctuations of climatological variables (e.g. Sturrock, Diaw, Talla, Niang, Piau, Capron, 2001, Liang, Seto, Remais, Zhong, Yang, Hubbard, Davis, Gu, Qiu, Spear, 2007) – most notably rainfall/temperature in tropical/temperate areas (McCreesh and Booth, 2013) – and environmental conditions (as in the case of fluctuating transmission risk during floods; e.g. Wu et al., 2008), both of which can remarkably influence snail population ecology (e.g. Chandiwana, Christensen, Frandsen, 1987, Woolhouse, Etard, Dietz, Ndhlovu, Chandiwana, 1998, Sturrock, Diaw, Talla, Niang, Piau, Capron, 2001). Water-contact patterns can also vary over time as a response to seasonal changes in human activities related, for instance, to agriculture and farming (e.g. Spear, Zhong, Mao, Hubbard, Birkner, Remais, Qiu, 2004, Liang, Seto, Remais, Zhong, Yang, Hubbard, Davis, Gu, Qiu, Spear, 2007). As a result of seasonal environmental forcing, transmission intensity and infection prevalence in both human and snail hosts are often observed to follow seasonal patterns (e.g. Chandiwana, Christensen, Frandsen, 1987, Woolhouse, Watts, Chandiwana, 1989, Sturrock, Diaw, Talla, Niang, Piau, Capron, 2001).

Heterogeneity has been accounted for in mathematical models for schistosomiasis transmission since long. A seminal contribution in this respect was given by Barbour (1978), who elaborated on Macdonald’s (1965) basic model, and extended it to account for individual variations in water contact patterns and source heterogeneity. In particular, Barbour derived an expression for the threshold parameter controlling disease transmission and concluded that heterogeneities in water contacts can favor endemicity. Later works focused on the role played by multiple water sources and individual heterogeneity (Woolhouse, Etard, Dietz, Ndhlovu, Chandiwana, 1998, Woolhouse, Watts, Chandiwana, 1991), site-specific environmental features (Liang, Maszle, Spear, 2002, Liang, Spear, Seto, Hubbard, Qiu, 2005), and the interplay between local-scale heterogeneities and spatial coupling mechanisms (Gurarie and King, 2005) in schistosomiasis transmission dynamics, with a special emphasis for the integration of field data and the analysis of strategies for disease control. Recent modeling studies have also focused on spatial heterogeneity arising from the pathogens’ dispersal mechanisms (such as human mobility for adult schistosomes and hydrological transport for their larval stages), as well as on the implications for parasite endemism (e.g. Gurarie, Seto, 2009, Perez-Saez, Mari, Bertuzzo, Casagrandi, Sokolow, De Leo, Mande, Ceperley, Frohelich, Sou, Karambiri, Yacouba, Maiga, Gatto, Rinaldo, 2015, Ciddio, Mari, Sokolow, De Leo, Gatto, Casagrandi, 2017). Although less frequently than transmission/spatial heterogeneity, temporal heterogeneity in schistosomiasis transmission has sometimes been also accounted for in both applied (e.g. Liang, Maszle, Spear, 2002, Liang, Spear, Seto, Hubbard, Qiu, 2005, Remais, 2010, Gurarie, King, Yoon, Alsallaq, Wang, 2017) and theoretical (Zhang, Gao, Cao, 2014, Ciddio, Mari, Gatto, Rinaldo, Casagrandi, 2015) studies.

In this work we aim to provide a comprehensive – yet as-simple-as-possible – account of the major sources of heterogeneity that can be relevant to schistosomiasis transmission. To that end, we make use of suitable extensions of the seminal Macdonald’s (1965) model, whose main characteristics are briefly reviewed in the next section. Then, we analyze a general transmission model (after Barbour, 1978) accounting for heterogeneities in both the human host population (expressed as differential exposure/contamination risk associated with different sub-groups within a community) and in the available water sources (as resulting from specific water contact patterns and the distribution of the snail host population). A threshold condition for the multi-group/multi-source model is worked out based on the dominant eigenvalue of a generalized reproduction matrix accounting for multiple sources of heterogeneity. The cases of heterogeneity in the human host population and in the available water sources are then analyzed separately through simple numerical examples, so as to single out the specific effects of the different sources of heterogeneity on schistosomiasis transmission dynamics. The case of spatial heterogeneity (which can be thought of as a special combination of mixed host/source heterogeneity) is also analyzed in some detail. Finally, a seasonally-forced version of the basic Macdonald’s (1965) model is studied, and conditions for long-term parasite establishment in periodic environments are obtained. A discussion about the most important epidemiological consequences of heterogeneity in schistosomiasis dynamics, with special focus on application to field data and disease control, closes the paper.

## 2 The basic homogeneous model

The simplest model for schistosomiasis was proposed by Macdonald in 1965. It describes transmission dynamics through two state variables, namely the average parasite burden in the human population (*P*) and the prevalence of infection in snails (*Y*). Population dynamics are neglected in human and snail hosts, assuming instead demographic equilibrium for both. Also, the model does not include the dynamics of cercariae and miracidia, whose abundances are considered to be proportional to *Y* and *P*, respectively. The model thus reads as follows:

(1) $$\begin{array}{ccc} \frac{dP}{dt} & = & \beta NY-\gamma P\\ \frac{dY}{dt} & = & \chi HP(1-Y)-\mu Y, \end{array}$$

where *β* is the snail-to-human transmission rate, *N* is the abundance of the snail population, *γ* is the mortality rate of adult parasites in human hosts, *χ* is the human-to-snail transmission rate, *H* is the abundance of the human population and *μ* is rate at which infected snails die and are replaced by uninfected ones. The parameters *γ* and *μ* can be evaluated as the inverse of the average lifespans of adult worms and infected snails (around five years and two months, respectively; see e.g. Feng et al., 2004), whereas *β* and *χ* represent aggregated parameters accounting for several epidemiological and socioeconomical processes.

Model (1) has two steady-state solutions. The first one is the so-called disease-free equilibrium (DFE), i.e. a state of the system in which the parasite is not present (thus $\bar{P}=0$ and $\bar{Y}=0$). The second one is the endemic equilibrium (EE), i.e a state of the system in which parasite transmission is permanent, namely

$$\bar{P}=\frac{\beta N\chi H-\gamma \mu }{\gamma \chi H},\;\bar{Y}=\frac{\beta N\chi H-\gamma \mu }{\beta N\chi H}.$$

**Fig. 1:**
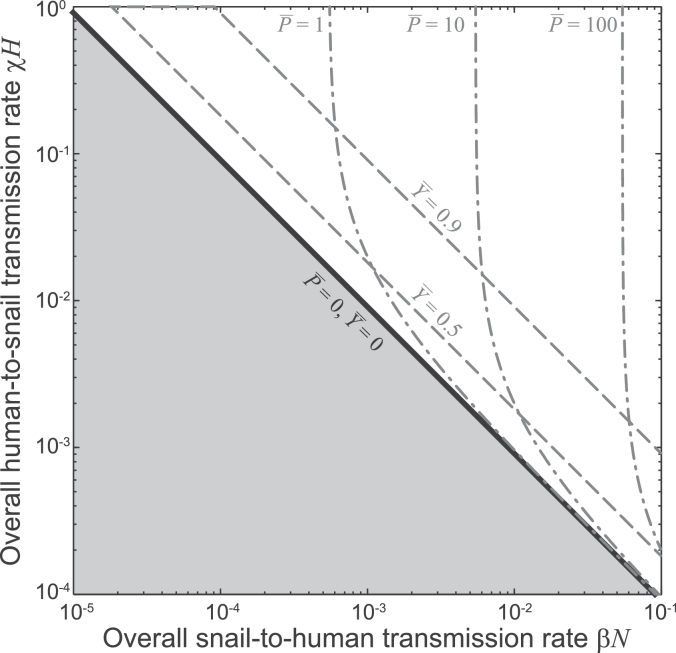
Equilibria of Macdonald’s (1965) homogeneous model (1). The DFE is stable if *r*_0_ < 1 (gray-shaded parameter combinations), while the EE is feasible and stable if *r*_0_ > 1. The black solid line indicates the transcritical bifurcation through which the two equilibria collide and exchange stability (endemicity boundary, $r_{0}=1$). The gray dashed curves are contour lines for the prevalence of infected snails, while the gray dash-dotted curves refer to the average worm burden in human hosts. Parameter values: $\gamma =5.5\cdot 10^{-4},$$\mu =1.7\cdot 10^{-2}$. All rates are expressed in [day${}^{-1}$].

By standard linear stability analysis arguments (Appendix A online), it can be shown that the DFE is stable if γμ−βNχH>0, also corresponding to the parameter region in which the EE is not feasible (i.e. characterized by negative components). This stability condition can be equivalently stated in terms of the so-called basic reproduction number

$$r_{0}=\frac{\beta N\chi H}{\gamma \mu },$$

i.e. the average number of established and reproductively mature offspring produced by a mature parasite during its lifetime in a population of uninfected hosts (Anderson and May, 1992). Specifically, if *r*_0_ < 1 the DFE is asymptotically stable, while the EE is unfeasible and unstable. Conversely, if *r*_0_ > 1 the DFE is unstable, while the EE is feasible and stable (see again Appendix A). Fig. 1 illustrates the basic properties of the equilibria of model (1). In particular, the shape of the contour lines for the average parasite burden in human hosts explains why preventing water contamination (i.e. decreasing the overall human-to-snail transmission rate, *χH*) might be less an effective measure for disease control (namely for decreasing $\bar{P}$) than preventing human water contact (i.e. decreasing the overall snail-to-human transmission rate, *βN*), as already suggested by Macdonald (1965) in his seminal work (see also Grimes et al., 2015, for discussion).

## 3 A general model for heterogeneous schistosomiasis transmission

The basic Macdonald’s (1965) transmission model (1) does not account for any of the different sources of heterogeneity that may be relevant to schistosomiasis transmission. Specifically, it does not include heterogeneities arising, for instance, from the presence of sub-groups in the human population, each of which may be endowed with different exposure/contamination rates, and/or from the availability of several water sources, whose characteristics (inclusive of local snail abundances) may possibly yield differential transmission risk. A general, multi-group model for schistosomiasis transmission was proposed by Barbour in 1978. Following his approach, we consider a community subdivided in *G* groups (e.g. according to age, sex or occupation, or to any other trait that may be relevant to schistosomiasis transmission) with access to *S* different water sources, all of which can possibly host snail populations. The model can be stated as

(2) $$\begin{array}{ccc} \frac{dP_{g}}{dt} & = & \beta \sum_{s=1}^{S}E_{gs}N_{s}Y_{s}-\gamma P_{g}\\ \frac{dY_{s}}{dt} & = & \chi \sum_{g=1}^{G}C_{gs}H_{g}P_{g}\left(1-Y_{s}\right)-\mu Y_{s}, \end{array}$$

where the subscript *g* [*s*] indicates variables or parameters pertaining to different human groups [water sources], while *E_gs_* [*C_gs_*] represents one entry of the human exposure [contamination] matrix, which describes the specific snail-to-human [human-to-snail] transmission risk associated with people from group *g* being in contact with water at source *s*. Clearly, a fully mechanistic description of the interwined processes that ultimately determine actual exposure/contamination risk for humans may be – at best – impractical. For the sake of simplicity, we thus describe human exposure/contamination as the product of three terms each, namely

$$E_{gs}=\epsilon_{g}^{E}\omega_{gs}\phi_{s}^{E}\;\mathrm{and}\;C_{gs}=\epsilon_{g}^{C}\omega_{gs}\phi_{s}^{C}\;,$$

in which $\epsilon_{g}^{E}$ [$\epsilon_{g}^{C}$] is a group-specific relative exposure [contamination] risk, *ω_gs_* represents the fraction of water contacts occurring at source *s* for people belonging to group *g* (so that 0 ≤ *ω_gs_* ≤ 1 and $\sum_{s}\omega_{gs}=1$) and $\phi_{s}^{E}$ [$\phi_{s}^{C}$] is a source-specific relative factor contributing to exposure [contamination]. Group-specific scores reflect differences in transmission risk related to demographic and socioeconomic traits. As an example, groups consisting prevalently of fishermen vs. blue- or white-collar workers may be endowed with different values of $\epsilon_{g}^{E}$ and $\epsilon_{g}^{C}$. Source-specific scores, instead, quantify differential transmission risk associated with the environmental characteristic of the water bodies. For instance, a shallow ephemeral pond may have intrinsically different values of $\phi_{s}^{E}$ and $\phi_{s}^{C}$ than a permanent river site. From a technical perspective, we assume that the union of the graphs associated with the exposure and contamination matrices ($E=[E_{gs}]$ and $C=[C_{gs}],$ respectively) is strongly connected, so as to prevent human groups and/or water sources from possibly being isolated as far as water contact is concerned.

Equilibrium solutions of model (2) include a DFE, in which ${\bar{P}}_{g}=0$ for all groups and ${\bar{Y}}_{s}=0$ for all sources, and an EE, whose components can be determined analytically only in some particular cases (Barbour, 1978). It can be shown (Appendix B) that the stability of the DFE of model (2) depends on the generalized reproduction matrix

$$R=r_{0}C^{T}hEn,$$

in which **h** and **n** are diagonal matrices whose non-zero elements correspond, respectively, to the fraction $h_{g}=H_{g}/H$ of the total human host population (*H*) belonging to each sub-group ($\sum_{g=1}^{G}h_{g}=1$) and to the fraction $n_{s}=N_{s}/N$ of the overall snail population (*N*) that can be found at each water site ($\sum_{s=1}^{S}n_{s}=1$), and the superscript *T* indicates matrix transposition. Specifically, the DFE of model (2) is asymptotically unstable if the dominant eigenvalue of matrix **R** ($R_{0}^{GS}=\lambda_{\max}\left(R\right),$ where the superscript *GS* indicates that the threshold parameter refers to a system with *G* human groups and *S* water sources) is larger than one, stable otherwise. This condition thus generalizes the analogous threshold based on the basic reproduction number *r*_0_ that is found for the basic Macdonald (1965) model (see Barbour, 1978; Gurarie and King, 2005). If $R_{0}^{GS}>1,$ numerical simulations of model (2) show that the EE is stable and characterized by strictly positive components.

In general, establishing parasite invasion/establishment conditions in the presence of transmission heterogeneity requires the numerical evaluation of $R_{0}^{GS}$. In the next sections we analyze two important special cases of model (2) for which the threshold parameter can be derived analytically.

### 3.1 Group heterogeneity: *G* human groups, one water source

We first analyze the case in which *G* ( > 1) sub-groups can be identified in a human population with access to one single water source (S=1). In this case, $\omega =\left[\omega_{gs}\right]=i_{G},$ where ****i_G_**** is a column vector of length *G* with all elements equal to one. With no loss of generality, we can assume $\phi_{s}^{E}=\phi_{s}^{C}=1,$ as changes in the source-specific exposure/contamination risk factors can be absorbed – with one water source – into the transmission rates *β* and *χ*. To allow a fair comparison with the results of the homogeneous model (1), we assume that the total human population abundance evaluated over all groups and the average exposure/contamination risk of the heterogeneous multi-group community are the same of an equivalent homogeneous community (i.e. $\sum_{g}h_{g}=1$ and $\sum_{g}h_{g}\epsilon_{g}^{E}=\sum_{g}h_{g}\epsilon_{g}^{C}=1$).

In the presence of group heterogeneity, the DFE is unstable, thus allowing for parasite invasion and long-term establishment in the community, if

$$R_{0}^{G1}=r_{0}\sum_{g=1}^{G}h_{g}\epsilon_{g}^{E}\epsilon_{g}^{C}>1,$$

(details in Appendix C). As already noted by Woolhouse et al. (1998), under the additional hypothesis that for each group the relative exposure risk is equal to the relative contamination risk ($\epsilon_{g}^{E}=\epsilon_{g}^{C}=\epsilon_{g}$), it is possible to show that heterogeneous multi-group communities sharing one water source cannot be less prone to long-term parasite establishment than homogeneous ones, i.e. that $R_{0}^{G1}\geq r_{0},$ all else being equal (see again Appendix C). Indeed, $R_{0}^{G1}=r_{0}$ only in the case of a homogeneous multi-group community ($\epsilon_{g}=1$ for all groups). Note that the simplification $\epsilon_{g}^{E}=\epsilon_{g}^{C}$ may indeed be reasonable for a disease, like schistosomiasis, in which exposure and contamination are both tightly related to water contact (Chandiwana, Woolhouse, 1991, Woolhouse, Etard, Dietz, Ndhlovu, Chandiwana, 1998, Gurarie, Seto, 2009). People spending more time at water sources where they can be exposed to the pathogen are also more likely to contribute to water contamination with their excreta. Therefore, the same behavior that increases exposure risk is also likely to increase contamination risk.

If $R_{0}^{G1}>1,$ the DFE is unstable and endemic schistosomiasis transmission is possible (stable EE). Three noteworthy results emerge for the EE of a heterogeneous multi-group community with one water source (details in Appendix C): a) the prevalence of infected snails cannot be smaller than in an equivalent homogeneous community (${\bar{Y}}^{G1}\geq \bar{Y}$); b) even some groups whose exposure/contamination risk is not larger than that of an equivalent homogeneous community (ϵ_*g*_ ≤ 1) may have a parasite load not smaller than that of an equivalent homogeneous community (${\bar{P}}_{g}^{G1}\geq \bar{P}$ for ϵ_*g*_ ≥ ϵ^⋆^, with ϵ^⋆^ ≤ 1); and c) the average parasite burden is not smaller than in an equivalent homogeneous community ($\sum_{g}h_{g}{\bar{P}}_{g}^{G1}\geq \bar{P}$). These three results depend on the fact that $\sum_{g}h_{g}\epsilon_{g}^{2}\geq 1$ in a heterogeneous multi-group community with one water source.

#### 3.1.1 The case of two groups

To elucidate the implications of heterogeneity associated with differential transmission risk, we analyze the simplest case of between-group heterogeneity, namely a community in which human hosts can be divided in two sub-populations (i.e. G=2), and where for each group the relative exposure risk is equal to the relative contamination risk ($\epsilon_{g}^{E}=\epsilon_{g}^{C}=\epsilon_{g}$). Let *f* be the fraction of human hosts belonging to the higher-risk group (say group 1, $h_{1}=f$ and $h_{2}=1-f,$ 0 < *f* < 1) and let *k* be the relative exposure/contamination risk of group 1 compared to group 2 ($\epsilon_{1}/\epsilon_{2}=k,$ *k* ≥ 1). To bettere contrast the effects of heterogeneous vs. homogeneous transmission, it is convenient to impose that the average exposure/contamination risk in the heterogeneous two-group community is the same as in the homogeneous one, that is $h_{1}\epsilon_{1}+h_{2}\epsilon_{2}=1,$ thus obtaining

$$\epsilon_{1}=\frac{k}{fk-f+1}\;\mathrm{and}\;\epsilon_{2}=\frac{1}{fk-f+1}.$$

**Fig. 2:**
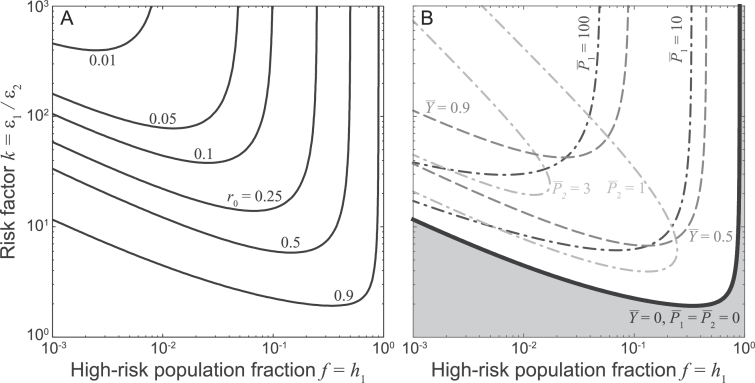
Analysis of a two-group community with access to a single water source (model (2) with G=2 and S=1). The two groups differ for their relative abundance (*h*_1_ and *h*_2_, with $h_{1}+h_{2}=1$) and intrinsic transmission risk (ϵ_1_ and ϵ_2_). A) Endemicity boundaries: parasite establishment is possible ($R_{0}^{21}>1$) for parameter combinations lying above the bifurcation curves, which correspond to $R_{0}^{21}=1$ and are obtained for different values of the basic reproduction number *r*_0_ of an equivalent homogeneous community (labels). B) State variables at the stable equilibrium: the dashed curves are contour lines for the prevalence of infected snails, while the dash-dotted curves refer to the average worm burden in human hosts (dark gray: high-risk group; light gray: low-risk group). The DFE is stable if $R_{0}^{21}<1$ (gray-shaded parameter combinations), while the EE is feasible and stable if $R_{0}^{21}>1$. The black solid line indicates the endemicity boundary $R_{0}^{21}=1$. In panel B, the overall exposure (*βN*) and contamination (*χH*) rates are assumed to be equal, and their value is set so as to match that of a homogeneous community endowed with a basic reproduction number $r_{0}=0.9$ (thus, $\beta N=\chi H=\sqrt{r_{0}\gamma \mu }$). Other parameters as in Fig. 1.

A few quantitative examples can help elucidate to what extent a two-group heterogeneous community can be more prone to the establishment of endemic schistosomiasis transmission than an equivalent homogeneous community. For instance, if a small fraction of the population (say 10%) is characterized by relatively large exposure/contamination risk (say 10 times higher) compared to the rest of the population, then $R_{0}^{21}\approx 3r_{0}$. If heterogeneity is even more pronounced (say 0.5% of the population with a 500-fold higher infection risk), then the parasite can establish in the whole community (i.e. also in the lower-risk group, albeit with a low average burden) even if *r*_0_ is as low as  ≈ 0.01. Fig. 2A generalizes these results and shows that a common feature of heterogeneous multi-group communities is sub-threshold endemic transmission (van den Driessche and Watmough, 2002), i.e. the long-term establishment of the parasite ($R_{0}^{21}>1$) under conditions that would prevent it in an equivalent homogeneous community (*r*_0_ < 1, for which $\bar{P}=0$ and $\bar{Y}=0$). Panel B of Fig. 2 shows the steady-state components of the EE in a heterogeneous two-group community in the case of sub-threshold transmission ($r_{0}=0.9$). The three theoretical results described above (namely, ${\bar{Y}}^{21}\geq \bar{Y},$${\bar{P}}_{2}^{21}\geq \bar{P}$ for ϵ_2_ ≤ 1, and $\sum_{g}h_{g}{\bar{P}}_{g}^{21}\geq \bar{P}$) are immediately recovered. Furthermore, we note that both the average parasite burden in the high-risk group and the prevalence of infected snails can attain very large values in case of pronounced heterogeneity. Not surprisingly, the average parasite burden in the low-risk group can be much (indeed, *k* times) smaller than in the high-risk one; however, because of the different group sizes, most parasites may actually be hosted within the members of the low-risk group.

More complex scenarios, i.e. describing communities with several (possibly many, *G* ≫ 2) risk groups relative to exposure and/or contamination risk and a single water source are discussed in Appendix C.

### 3.2 Source heterogeneity: homogeneous human population, *S* water sources

We now turn to the study of a homogeneous human community (G=1) with access to a set of *S* (>1) different water sources. In this case, matrix ******ω****** reduces to a row vector $u=[\omega_{1}\cdots \omega_{S}],$ whose elements *ω_s_* represent the fraction of water contacts that take place at each source *s*. With no loss of generality, we can set $\epsilon_{g}^{E}=\epsilon_{g}^{C}=1,$ as changes in group-specific exposure/contamination risk factors could be incorporated – with just one group – into the transmission rates *β* and *χ*. For the sake of parameter parsimony, and to avoid possible confounding effects, we also assume $\phi_{s}^{E}=\phi_{s}^{C}=1,$ i.e. that water sources share the same characteristics leading to exposure/contamination, including their accessible water volume. On the other hand, heterogeneity is granted by the distribution of the overall snail population among different water sources, as well as by the distribution of water contacts. To allow a fair comparison with the homogeneous case of model (1), the total snail abundance and the overall frequency of human-water contacts evaluated over all water points are assumed to be the same as in an equivalent homogeneous system (i.e. $\sum_{s}n_{s}=\sum_{s}\omega_{s}=1$).

In the presence of source heterogeneity, the DFE is unstable and endemic transmission is possible if

$$R_{0}^{1S}=r_{0}\sum_{s=1}^{S}n_{s}\omega_{s}^{2}>1.$$

Details are reported in Appendix D, where it is also shown that $R_{0}^{1S}\leq r_{0}$ for all water contact and snail distribution patterns. Therefore, as already noted by Woolhouse et al. (1991), a dilution effect induced by the parcelling of the overall snail population and the human water contacts among the available sources exists. This dilution effect can decrease the risk of schistosomiasis endemicity, all else being equal. If $R_{0}^{1S}>1,$ the DFE is unstable. In this case, although a compact analytical expression for its coordinates is not readily available, the stability of the EE can easily be tested via numerical simulation.

#### 3.2.1 The case of two water sources

**Fig. 3:**
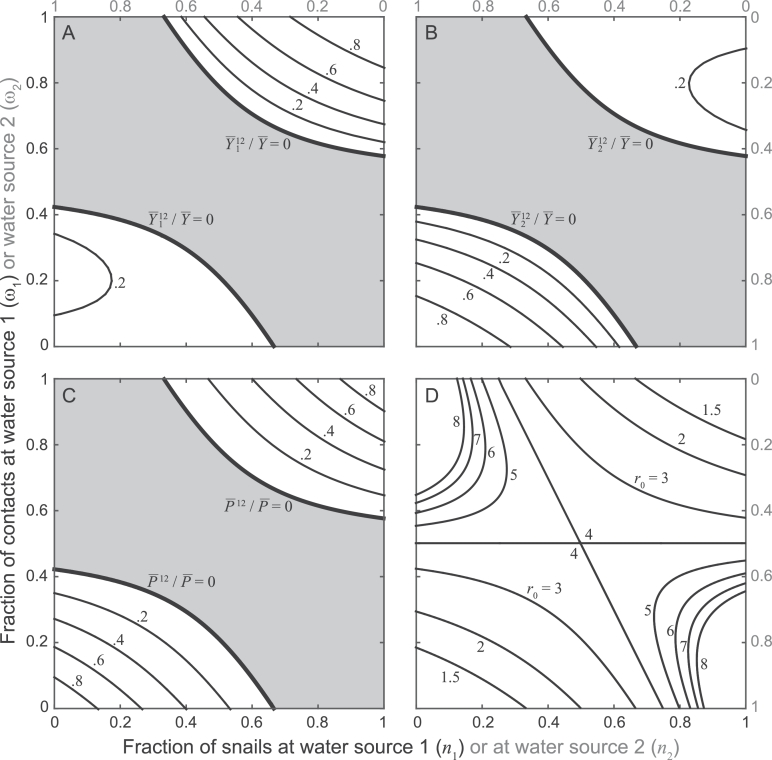
Analysis of a single-group community with access to two water sources (model (2) with G=1 and S=2). The two sources differ for the relative abundance of snails they host (*n*_1_, bottom axes, and *n*_2_, top axes, with $n_{1}+n_{2}=1$) and the frequency of human-water contacts (*ω*_1_, left axes, and *ω*_2_, right axes, with $\omega_{1}+\omega_{2}=1$). A) Steady-state prevalence of infection in snails at water source 1 (${\bar{Y}}_{1}^{12}$) divided by the steady-state prevalence of infected snails in an equivalent homogeneous community ($\bar{Y}$) with $r_{0}=3$ ($\beta N=\chi H=\sqrt{r_{0}\gamma \mu },$ other parameters as in Fig. 1). Black curves are contour lines of ${\bar{Y}}_{1}^{12}/\bar{Y}$. B) As in panel A, for the steady-state prevalence of infection in snails at water source 2 (${\bar{Y}}_{2}^{12}$). Black curves are contour lines of ${\bar{Y}}_{2}^{12}/\bar{Y}$. C) As in panel A, for the steady-state average parasite burden in human hosts (${\bar{P}}^{12}$). Black curves are contour lines of ${\bar{P}}^{12}/\bar{P}$ (where $\bar{P}$ is the steady-state average parasite burden in the equivalent homogeneous community). The 0-level lines in panels A–C (thick lines) correspond to the endemicity boundary $R_{0}^{12}=1,$ while the gray-shaded regions correspond to parameter combinations for which the DFE of the heterogeneous model is stable ($R_{0}^{12}<1$). D) Endemicity boundaries in the heterogeneous community ($R_{0}^{12}=1$) for different values of the basic reproduction number of an equivalent homogeneous community (labels). Parasite establishment is possible ($R_{0}^{12}>1$) for parameter combinations lying within the region(s) including the homogeneous cases $n_{1}=\omega_{1}=0$ and/or $n_{1}=\omega_{1}=1$.

To better analyze parasite establishment in a community with access to *S* water sources we refer to the simplest setting, namely the case in which S=2. Fig. 3 illustrates the dilution effect induced by the presence of multiple water sources, in terms of both infection intensity and endemicity risk. Specifically, panels A–C shows a comparison between the equilibrium values of the state variables describing the heterogeneous two-source community at equilibrium (obtained via numerical simulation) and the equivalent homogeneous case of model (1). Both the prevalence of infection in snails and the average parasite burden in human hosts are found to be smaller in the two-source community. In particular, super-threshold parasite extinction (i.e. occurring for *r*_0_ > 1) is possible if a large share of water contacts occur at a water source hosting a small fraction of the overall snail population (or equivalently, if a small share of water contacts occur at a water source hosting a large fraction of the overall snail population; gray-shaded parameter combinations). Panel D of Fig. 3 shows instead that $R_{0}^{1S}$ approaches *r*_0_ only if *n*_1_ and *ω*_1_ are either both large or both small, i.e. in cases in which heterogeneity is practically negligible. Conversely, $R_{0}^{1S}$ progressively decreases compared to *r*_0_ as more water contacts are made at relatively snail-free sources (i.e. if *n*_1_ is large and *ω*_1_ is small or, viceversa, if *n*_1_ is small and *ω*_1_ is large). In case of uniform snail distribution ($n_{1}=n_{2}=1/2$), endemicity risk decreases (higher values of *r*_0_ are needed for the DFE to become unstable) as the heterogeneity of human-water contact also decreases ($|\omega_{1}-\omega_{2}|\to 0$).

Similar analyses can be performed with reference to more complex case studies, i.e. communities with access to several (possibly many, *S* ≫ 1) water sources. Some examples are discussed in Appendix D.

### 3.3 Spatial heterogeneity: multiple goups and water sources

Model (2) can be used to study the effects of heterogeneity also in a spatially-explicit setting. The *G* human groups could in fact belong to different villages; also, they could have access to a set of *S* water sources arranged in a network endowed with a well-defined spatial structure (see e.g. Gurarie, King, 2005, Gurarie, Seto, 2009, Perez-Saez, Mari, Bertuzzo, Casagrandi, Sokolow, De Leo, Mande, Ceperley, Frohelich, Sou, Karambiri, Yacouba, Maiga, Gatto, Rinaldo, 2015, Ciddio, Mari, Sokolow, De Leo, Gatto, Casagrandi, 2017). In this case, the water contact matrix ******ω****** can be thought of as a human mobility matrix, at least as far as water use is concerned. Here we limit our attention to the simplest case of spatial heterogeneity, namely a metacommunity with two sub-groups (G=2) living in two geographically distinct locations, each of which has one preferential water source (S=2). If we assume that water contacts occur not only at the source that is closest to the home site, but also when people travel between the two sites, the water contact matrix can be defined as

$$\omega =\left[\begin{array}{cc} 1-m_{1} & m_{1}\\ m_{2} & 1-m_{2} \end{array}\right],$$

where the anti-diagonal elements represent the fraction of water contacts occurring away from the home site. The only spatial coupling process considered in the model is human mobility, which seems to be appropriate if water sources are not hydrologically connected, or if they are quite distant from each other.

**Fig. 4:**
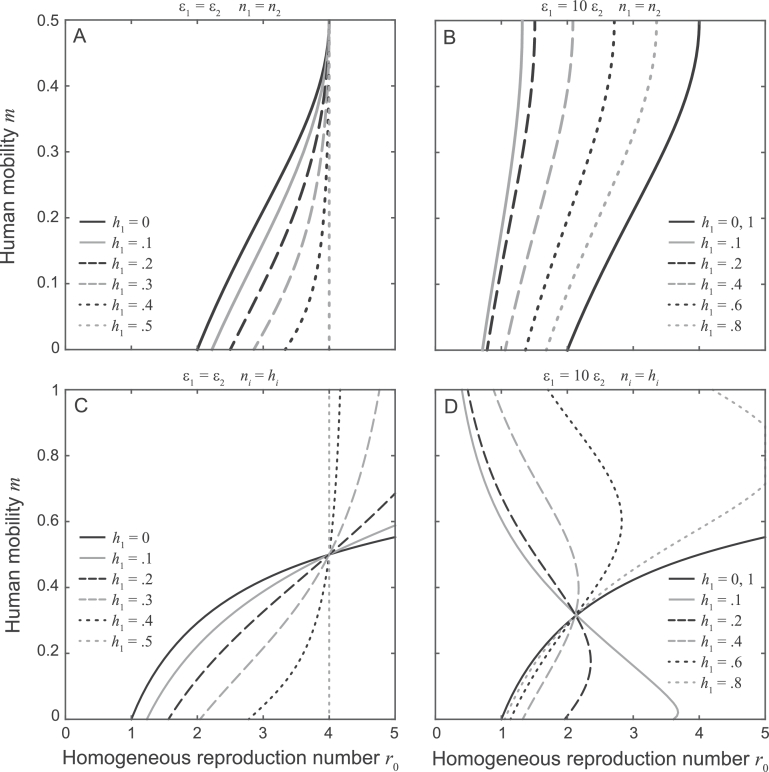
Endemicity boundaries in a spatially heterogeneous metacommunity with two human groups living in separate locations, each with one preferential water source (model (2) with G=2 and S=2). The groups may differ for their relative abundance (*h*_1_ and *h*_2_, with $h_{1}+h_{2}=1$) and intrinsic transmission risk (ϵ_1_ and ϵ_2_), while the two sources may differ for the relative abundance of snails they host (*n*_1_ and *n*_2_, with $n_{1}+n_{2}=1$) and the frequency of human-water contacts (for each community, the fraction of contacts at the farthest water point is *m*, while 1−m is the fraction of contacts at the home site). Parasite establishment is possible ($R_{0}^{22}>1$) on the right of the bifurcation curves, which correspond to $R_{0}^{22}=1$ and are obtained for different spatial distributions of the human host population (legend). A) Spatially homogeneous transmission risk ($\epsilon_{1}/\epsilon_{2}=1$) and snail population distribution ($n_{1}=n_{2}=1/2$). B) As in panel A, with spatially heterogeneous transmission risk ($\epsilon_{1}/\epsilon_{2}=10$). C) As in panel A, with spatially heterogeneous snail abundance ($n_{1}=h_{1}$). D) Spatially heterogeneous transmission risk ($\epsilon_{1}/\epsilon_{2}=10$) and snail population distribution ($n_{1}=h_{1}$).

To allow a fair comparison with the (spatially) homogeneous case, we use the same human and snail total population abundances as in the homogeneous case. Furthermore, we assume that $\epsilon_{i}^{E}=\epsilon_{i}^{C}=\epsilon_{i}$ and $\phi_{i}^{E}=\phi_{i}^{C}=1$ for both i=1,2, and define ϵ_1_ and ϵ_2_ so that the average exposure/contamination risk in the (spatially) heterogeneous metacommunity is the same as in the homogeneous community. Finally, we set $m_{1}=m_{2}=m$ for the sake of simplicity. The stability of the DFE ([0 0 0 0]^*T*^) can be assessed through the evaluation of the dominant eigenvalue of matrix ****R****, which can be easily performed numerically. However, in spite of its simplicity, the spatially explicit version of the transmission model still holds many degrees of freedom, namely: the values of the average exposure and contamination rates (whose product concurs to the definition of the basic reproduction number in the equivalent homogeneous community); the level of human mobility, as measured by the fraction of water contacts occurring away from the home site; the distributions of the human/snail populations in the two sites; and the spatial heterogeneity of exposure/contamination risk. This makes a complete study of the endemicity threshold quite cumbersome. Fig. 4 thus reports the analysis of parasite establishment conditions for four selected settings.

In the first example (Fig. 4A), exposure/contamination risk is the same in the two sites and the snail population is uniformly distributed between the two water sources. The endemicity boundary is symmetric with respect to $h_{1}=h_{2}=1/2$ and m=1/2, which means that the values of $R_{0}^{22}$ evaluated for $h_{i}=\bar{h}$ or $h_{i}=1-\bar{h},$ and for $m=\bar{m}$ or $m=1-\bar{m}$ coincide. Therefore, only results obtained for 0 ≤ *h*_1_ ≤ 1/2 and 0 ≤ *m* ≤ 1/2 are shown. In this case, mobility shows a well-defined dilution effect, with increasing *m* values playing against long-term pathogen establishment. The endemicity boundary generally (i.e. except for m=1/2, corresponding to complete spatial mixing) moves rightward as the fraction of residents of site 1 increases from 0 to 1/2, which indicates that more homogeneous population distributions are less conducive to the establishment of endemic parasite transmission. For instance, if the total human population were uniformly distributed between the two sites, the parameters combinations yielding $R_{0}^{22}>1$ would give *r*_0_ > 4 (note that, in this case, each village/water source would host exactly half of the human/snail population of an equivalent homogeneous community).

The second example (Fig. 4B) accounts for spatial heterogeneity in terms of exposure/contamination risk. This might represent the (quite common) case of a village composed by different neighborhoods that share the same water contact points but are composed of a diversified human population, with people characterized by distinct socioeconomic traits. Such differences in demographic composition could indeed yield a correspondence between spatial and transmission heterogeneities, as mediated by different water-related behavior (which would most likely be preserved during movement, especially over relatively short spatial scales). For instance, Pinot de Moira et al. (2007) found an approximately 10-fold difference in the overall duration of water contacts (mostly related to fishing activities) between groups of adult males with different tribal backgrounds living in separated neighborhoods of a community in rural Uganda. Because of the spatial heterogeneity in exposure/contamination, the endemicity boundary is no longer symmetric about $h_{1}=h_{2}=1/2,$ while it still is about m=1/2. Therefore, only results obtained for 0 ≤ *m* ≤ 1/2 are shown. Interestingly, it turns out that $R_{0}^{22}$ is maximum compared to *r*_0_ for intermediate values of *h*_1_ (actually, for 0 < *h*_1_ < 0.2). In other words, the likelihood of endemicity is maximum if a relatively small fraction ( ≈ 10% in this example) of the human population lives in the village where the most-at-risk water source is located. Mobility is again associated with a dilution effect. For some population distributions and mobility levels (i.e. for $h_{1}=0.1$ and m=0.05), $R_{0}^{22}$ can indeed be larger than one even if *r*_0_ is not (sub-threshold parasite establishment), which suggests that the spatial heterogeneity of the transmission parameters can strongly favor endemic transmission also in the presence of human mobility.

The third example (Fig. 4C) differs from the first one in that snail population abundance is spatially heterogeneous. Specifically, the spatial distribution of snail hosts is assumed to be linked to that of the human population ($n_{i}=h_{i}$). This might be reasonable, for instance, should a positive feedback between larger human groups and larger snail populations exist, as it may be expected in rural contexts where man-made alterations of the environment (such as the construction of reservoirs and irrigation schemes for agricultural development) can contribute to creating new habitat for the snails (Steinmann et al., 2006). Because of the spatial heterogeneity of snail distribution, the endemicity boundary is no longer symmetric with respect to m=1/2, while it still is about $h_{1}=h_{2}=1/2$. Therefore, only results obtained for 0 ≤ *h*_1_ ≤ 1/2 are shown. High levels of human mobility are typically associated with parasite extinction (possibly except for $h_{1}=h_{2}=1/2$). It is also interesting to note that the endemicity boundary moves rightward for increasing values of $h_{1}=n_{1}$ only if *m* < 1/2, leftward otherwise (*m* > 1/2).

In the fourth example (Fig. 4D) both the exposure/contamination risk and the snail distribution are assumed to be spatially heterogeneous. As a result, the endemicity boundary is symmetric about neither $h_{1}=h_{2}=1/2$ nor m=1/2. The most interesting result pertaining to this case is the appearance of backward-bending endemicity boundaries. Of particular importance is the region identified by *m* < 1/2, where mobility is found to play a significant role. Specifically, for 0 < *h*_1_ < 1/2, $R_{0}^{22}$ is minimum for intermediate values of *m*. Therefore, depending on *h_i_* and *r*_0_, increasing human mobility can either hinder or promote endemic parasite transmission. With $h_{1}=0.2$ and $r_{0}=2,$ for example, parasite establishment is possible for m=0 or m=0.4, but not for m=0.2. Interestingly, even relatively low values of the ϵ_1_/ϵ_2_ ratio are sufficient to recover qualitatively similar results (e.g. $\epsilon_{1}/\epsilon_{2}=2$; see Appendix E for some examples). Finally, we note that cases in which more abundant snail populations are associated with less abundant human communities (e.g. $n_{i}=1-h_{i}$) might instead be found in different socioeconomic contexts and/or spatial scales, such as in the case of a spatially explicit setting encompassing both urban and rural environments. Note that results for this latter scenario can be recovered from the former’s, as the values of $R_{0}^{22}$ evaluated for $m=\bar{m}$ under the assumption $n_{i}=1-h_{i}$ would coincide with those evaluate for $m=1-\bar{m}$ with $n_{i}=h_{i}$.

## 4 A special case of heterogeneity: time-varying transmission

So far we have been dealing with heterogeneity arising from two basic factors, namely differential infection risk among different sub-groups within a human community, and/or differential transmission related to the distribution of water contacts and snail hosts among the available water sources – with spatial heterogeneity representing a particular (yet noteworthy) combination of these two factors. In all cases, we have (mostly) focused on the relationship between transmission heterogeneity and parasite invasion/establishment. One possible source of heterogeneity that is not accounted for in models (1) and (2) (hence also in the special cases analyzed above) is the temporal variability of the parameters that are relevant to schistosomiasis dynamics. Some seasonally-forced models for schistosomiasis transmission have recently been proposed. For instance, Zhang et al. (2014) analyzed a model in which the human population is split into susceptible/infected hosts, without accounting for the average parasite burden in the community, as it is instead customary in models for macroparasite transmission. For this reason, their approach lies somewhat outside Macdonald’s (1965) modeling framework. Ciddio et al. (2015), instead, proposed a Macdonald-like model accounting for the demographic dynamics of the snail population. They showed that the interplay between density dependence and temporal forcing may lead to complex (and realistic) transmission dynamics, yet they did not specifically focus on the conditions leading to endemic schistosomiasis transmission. Finally, other temporally-forced models (e.g. Liang, Maszle, Spear, 2002, Liang, Spear, Seto, Hubbard, Qiu, 2005, Remais, 2010, Gurarie, King, Yoon, Alsallaq, Wang, 2017) were mainly tailored to analyze real case studies, hence they are not fully amenable to theoretical investigation.

The effects of time-varying transmission on parasite invasion/establishment can be analyzed with the following time-varying version of model (1), i.e.

(3) $$\begin{array}{ccc} \frac{dP}{dt} & = & \tilde{\beta }\left(t\right)\tilde{N}\left(t\right)Y-\tilde{\gamma }\left(t\right)P\\ \frac{dY}{dt} & = & \tilde{\chi }\left(t\right)\tilde{H}\left(t\right)P\left(1-Y\right)-\tilde{\mu }\left(t\right)Y, \end{array}$$

where

$$\begin{array}{ccc} \tilde{\beta }\left(t\right) & = & \beta \left[1+\alpha_{\beta }\sin\left(\frac{2\pi }{\tau_{\beta }}\left(t+\psi_{\beta }\right)\right)\right]\;\mathrm{and}\\ \tilde{\chi }\left(t\right) & = & \chi \left[1+\alpha_{\chi }\sin\left(\frac{2\pi }{\tau_{\chi }}\left(t+\psi_{\chi }\right)\right)\right] \end{array}$$

are the seasonally varying snail-to-human and human-to-snail transmission rates,

$$\begin{array}{ccc} \tilde{N}\left(t\right) & = & N\left[1+\alpha_{N}\sin\left(\frac{2\pi }{\tau_{N}}\left(t+\psi_{N}\right)\right)\right]\;\mathrm{and}\\ \tilde{H}\left(t\right) & = & H\left[1+\alpha_{H}\sin\left(\frac{2\pi }{\tau_{H}}\left(t+\psi_{H}\right)\right)\right] \end{array}$$

are the seasonally varying population abundances of snails and humans, and, finally,

$$\begin{array}{ccc} \tilde{\gamma }\left(t\right) & = & \gamma \left[1+\alpha_{\gamma }\sin\left(\frac{2\pi }{\tau_{\gamma }}\left(t+\psi_{\gamma }\right)\right)\right]\;\mathrm{and}\\ \tilde{\mu }\left(t\right) & = & \mu \left[1+\alpha_{\mu }\sin\left(\frac{2\pi }{\tau_{\mu }}\left(t+\psi_{\mu }\right)\right)\right] \end{array}$$

are the seasonally varying mortality rates of adult parasites and infected snails, respectively. In the above expressions, *α_x_* represents the amplitude of seasonal fluctuations (0 ≤ *α_x_* ≤ 1), *τ_x_* is the period of the oscillations and *ψ_x_* represents the phase of environmental fluctuations (*x* ∈ {*β, χ, N, H, γ, μ*}).

To simplify the analysis of model (3), we assume that the fluctuations of the transmission rates are mostly associated with seasonal variations of the human-water contact rate, so that $\tilde{\beta }\left(t\right)$ and $\tilde{\chi }\left(t\right)$ are characterized by the same amplitude ($\alpha_{\beta }=\alpha_{\chi }=\alpha_{\omega }$) and phase ($\psi_{\beta }=\psi_{\chi }=\psi_{\omega }$). Also, because of the long lifespan of schistosomes within human hosts, it is reasonable to neglect fluctuations of the mortality rate of adult parasites ($\alpha_{\gamma }=0$). Seasonal variations of the mortality rate of infected snails are not considered either ($\alpha_{\mu }=0$), as they are better addressed in schistosomiasis transmission models that include a detailed description of the snail host ecology (see e.g. Ciddio, Mari, Gatto, Rinaldo, Casagrandi, 2015, Sokolow, Huttinger, Jouanard, Hsieh, Lafferty, Kuris, Riveau, Senghor, Thiam, N’Diaye, Sarr Faye, De Leo, 2015, Gurarie, King, Yoon, Alsallaq, Wang, 2017). The period of oscillations is assumed to be one year for all the seasonal environmental signals ($\tau_{x}=\tau =365$ [d], *x* ∈ {*β, χ, N, H*}). Without loss of generality, we can also set $\psi_{\omega }=0$ and study the effects of possible lags *ψ_x_* between the phase of the transmission rates and the other parameters’ (0 ≤ *ψ_N_, ψ_H_* ≤ *τ*). A simplified version of model (3) incorporating all these assumptions is given in Appendix F.

Like in model (1), the DFE of model (3) is a state in which $\bar{P}=0$ and $\bar{Y}=0$. However, determining the stability properties of the DFE (or, equivalently, the conditions under which the parasite can establish in the community) is relatively more complex in this case, because Floquet theory (instead of basic linear stability analysis) has to be applied (see e.g. Bacaër and Guernaoui, 2006; Klausmeier, 2008; Bittanti, Colaneri, 2009, Mari, Casagrandi, Bertuzzo, Rinaldo, Gatto, 2014). Specifically, the DFE is unstable (thus allowing parasite invasion and the establishment of periodic tramsmission cycles) if and only if $R_{0}^{F},$ the maximum Floquet multiplier of system (3) linearized in a neighborhood of the DFE, is larger than one. Floquet multipliers are defined as the eigenvalues of the monodromy matrix ****M****(*τ*), which can be obtained by solving the matrix differential equation

$$\begin{array}{ccc} & & \frac{dM(t)}{dt}=J_{0}\left(t\right)\;M\left(t\right)=\left[\begin{array}{cc} -\tilde{\gamma }\left(t\right) & \tilde{\beta }\left(t\right)\tilde{N}\left(t\right)\\ \tilde{\chi }\left(t\right)\tilde{H}\left(t\right) & -\tilde{\mu }\left(t\right) \end{array}\right]\\ & & M\left(t\right)=\left[\begin{array}{cc} -\gamma & \tilde{\beta }\left(t\right)\tilde{N}\left(t\right)\\ \tilde{\chi }\left(t\right)\tilde{H}\left(t\right) & -\mu \end{array}\right]M\left(t\right) \end{array}$$

over one period (i.e. over 0 ≤ *t* ≤ *τ*), with the identity matrix as initial condition (see again Klausmeier, 2008). Note that $R_{0}^{F}$ is equal to *r*_0_ in the time-constant case ($\alpha_{x}=0$ for all *x*’s).

**Fig. 5:**
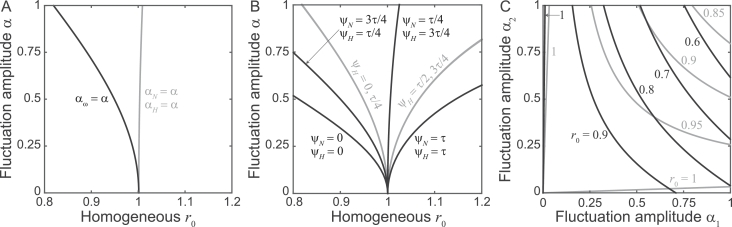
Endemicity boundaries in the presence of periodically forced transmission (model (3)). Long-term pathogen establishment is possible ($R_{0}^{F}>1$) on the right of the endemicity curves, which correspond to $R_{0}^{F}=1$. A) Periodic veriations of exposure/transmission risk (black: $\alpha_{\phi }=\alpha$ and $\alpha_{N}=\alpha_{H}=0$) or of the population abundance of either host species (gray: $\alpha_{\phi }=0$ and either $\alpha_{N}=\alpha$ or $\alpha_{H}=\alpha$). B) Coupled fluctuations of exposure/transmission risk and of the population abundance of either host species (black: $\alpha_{\phi }=\alpha ,$ and either $\alpha_{N}=\alpha$ and $\alpha_{H}=0$ or $\alpha_{H}=\alpha$ and $\alpha_{N}=0$), or of the population abundances of the two host species (gray: $\alpha_{\phi }=0,$$\alpha_{N}=\alpha_{H}=\alpha$ and $\psi_{N}=0$), for different phase shifts between the relevant periodic signals (labels). C) As in panel B, for synchronous coupled fluctuations with different amplitudes (black: $\alpha_{\phi }=\alpha_{1},$ and either $\alpha_{N}=\alpha_{2},$$\alpha_{H}=0$ and $\psi_{N}=0$ or $\alpha_{H}=\alpha_{2},$$\alpha_{N}=0$ and $\psi_{H}=0$; gray: $\alpha_{\phi }=0,$$\alpha_{N}=\alpha_{1},$$\alpha_{H}=\alpha_{2}$ and $\psi_{N}=\psi_{H}=0$). Results are shown for different values of the basic homogeneous reproduction number *r*_0_ (labels). In all panels, $\beta N=\chi H=\sqrt{r_{0}\gamma \mu }$; other parameters as in Fig. 1.

The results concerning parasite invasion in seasonal environments are reported in Fig. 5. Seasonal fluctuations can make the instability of the DFE either less or more likely, depending on the parameter (panel A) or combination of parameters (panels B and C) that is assumed to vary over time. Specifically, if the abundance of one of the two host populations fluctuates periodically (0 < *α_N_* ≤ 1 or 0 < *α_H_* ≤ 1, e.g. because of seasonal variations of snail demography, or human migrations linked to seasonal activities), then $R_{0}^{F}<r_{0}$. In these two cases, pathogen persistence requires larger average values of the time-varying parameters than in the time-constant case. On the contrary, $R_{0}^{F}>r_{0}$ if the human exposure/contamination varies seasonally (0 < *α_ω_* ≤ 1). In this latter case, endemic transmission is favored by seasonal forcing (Fig. 5A). Coupled fluctuations of the human-water contact rate and the population abundance of either snails or humans are associated with the largest deviations from the endemicity threshold $r_{0}=1$ of the time-homogeneous case. Phase shifts between the two periodic signals are of paramount importance to qualify such deviations: on the one hand, synchronous fluctuations ($\psi_{N}=0$ or $\psi_{H}=0,$ i.e. more frequent human-water contact when snails or human hosts are more abundant) promote sub-threshold endemicity, i.e. parasite invasion and establishment being possible for *r*_0_ < 1; on the other hand, periodic signals that are in antiphase with each other ($\psi_{N}=\tau /2$ or $\psi_{H}=\tau /2,$ e.g. more frequent human-water contact when snails or human hosts are less abundant) hinder endemicity, with parasite extinction being possible for *r*_0_ > 1. Similar results are obtained with coupled fluctuations of the two hosts’ population abundances, although with smaller deviations from the time-homogeneous case (Fig. 5B). The relative amplitude of fluctuations also matters in case of coupled seasonal forcing (Fig. 5C).

## 5 Discussion and conclusions

In this work we have analyzed a flexible modeling framework to describe schistosomiasis transmission dynamics in the presence of various sources of heterogeneity, including differential transmission risk linked to the demographic or socioeconomic traits of the human host population, availability of multiple water sources characterized by non-homogeneous water contacts and snail host distribution, spatially explicit water contact patterns, and temporal fluctuations of the eco-epidemiological parameters relevant to disease transmission. Overall, a clear picture emerges where heterogeneity plays a highly non-trivial role in the definition of epidemiological patterns. Specifically, between-group heterogeneity in transmission risk is typically associated with larger infection prevalence in snails and average parasite burden in humans, as well as with a higher likelihood of long-term parasite invasion. On the other hand, heterogeneity induced by the presence of different water sources may produce opposite effects, namely lower infection intensity in humans and snails, and lower chances of endemic parasite transmission compared to a homogeneous situation. All these results conform with and extend previous findings on heterogeneous schistosomiasis dynamics (Barbour, 1978, Woolhouse, Watts, Chandiwana, 1991, Woolhouse, Etard, Dietz, Ndhlovu, Chandiwana, 1998). Results concerning the spatially heterogeneous case reveal that the epidemiological implications of human mobility may be different in different contexts. Interestingly, there are cases in which the likelihood of endemic transmission is maximum for intermediate levels of human mobility. A similar scenario has been discussed in a recent modeling study of the geography of schistosomiasis transmission in Burkina Faso (Perez-Saez et al., 2015). Therefore, despite the highly simplified setting used in this work, our analysis suggests that the interplay between transmission heterogeneity and spatial heterogeneity can provide a theoretical background to results found in real or realistic case studies. Finally, the analysis of temporal heterogeneity in schistosomiasis transmission has revealed that the effects of seasonal forcing are context-dependent too. In fact, temporal heterogeneity can either promote or play against long-term parasite persistence in the community, depending on the eco-epidemiological parameters that are affected by environmental fluctuations.

**Fig. 6:**
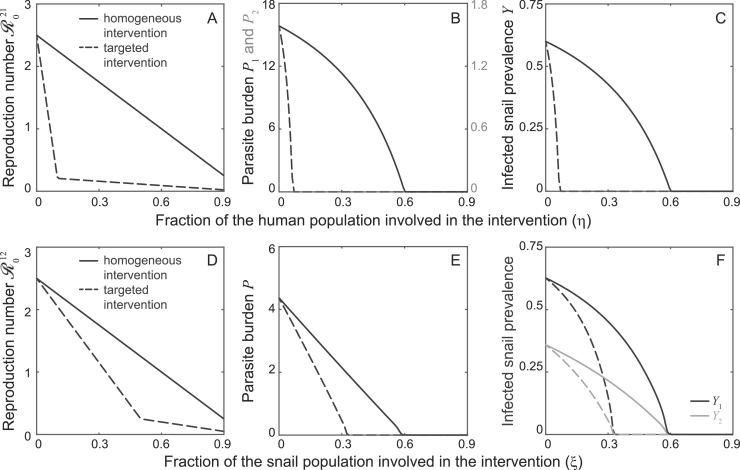
Analysis of exemplificative strategies for schistosomiasis control in the presence of heterogeneity. Top panels refer to the case of a two-group community with access to a single water source (as in Fig. 2 with f=0.1 and k=10). A) Reduction of the reproduction number $R_{0}^{21}$ for increasing shares of the human population being involved in the intervention. B) Reduction of the average parasite burden in the two sub-groups (left axis: high-risk group; right axis: low-risk group). C) Reduction of infected snail prevalence. Bottom panels refer to the case of a single community with access to two water sources (as in Fig. 3, with $\omega_{1}=3/4,$$\omega_{2}=1/4$ and $n_{1}=n_{2}=1/2$). D) Reduction of the reproduction number $R_{0}^{12}$ for increasing shares of the snail population being removed. E) Reduction of the average parasite burden. F) Reduction of infected snail prevalence at the two water sources. In all cases, βN=χH are set so that the reproduction number in the heterogeneous community is equal to 2.5 prior to the intervention. Other parameters as in Fig. 1. See text for details on the interventions.

Although these results are noteworthy *per se*, the greater goals of mathematical epidemiology lie in the applicability of theoretical tools to real case studies and in the pursuit of opportunities for disease control. As far as parasite control is concerned, heterogeneity associated with differential transmission risk (group heterogeneity) seems to be the most critical and targetable factor that unambiguously favors schistosomiasis transmission. In this case, in fact, the presence of sub-threshold endemic dynamics suggests that it may be very difficult to break transmission in highly heterogeneous human communities, unless suitable interventions aimed at the most-at-risk share of the population (e.g. children, fishermen, or women, in the case of schistosomiasis) are implemented. The prototypical case of a two-group community with access to one single water source can be used again to get a handle on the importance of tracking down heterogeneity to design effective disease control strategies. As an example, let us consider a community where, prior to any intervention, 10% of the population has a 10-fold transmission risk and $R_{0}^{21}=2.5$. Suppose that there exists some action (be it sustained chemoteraphy administration, improved hygiene or education; see e.g. Rollinson et al., 2013) by which a fraction *η* of the total population can be effectively removed from being susceptible to infection (independently of the risk group they belong to). Also, assume that the implementation of the interventions can be either evenly spread within the population or targeted (i.e. high-risk group members are prioritized). In the former case, the allocation of the population between the two risk groups does not change; in the latter, the high-risk share of the population is reduced to max(0,(f−η)/(1−η)). The top panels of Fig. 6 show that bringing $R_{0}^{21}$ below one, thus breaking transmission, would require treatment for 7% or 60% of the overall human population with targeted or untargeted interventions, respectively.

The dual case of heterogeneity associated with non-homogeneous water contact patterns and/or snail distributions (source heterogeneity) may be seen as relatively less critical, in general, because the dilution effect induced by the availability of multiple water sources typically hinders long-term parasite establishment. However, one could still investigate possible differences between targeted (priority is given to the most used and/or snail-rich water sources) and homogeneous snail removal (be it by means of focal molluscicides or biological control; see King, Bertsch, 2015, Sokolow, Huttinger, Jouanard, Hsieh, Lafferty, Kuris, Riveau, Senghor, Thiam, N’Diaye, Sarr Faye, De Leo, 2015). We refer to the prototypical case of a single well-mixed human community with access to two water sources. As an example, let us consider a community in which, prior to any intervention, the overall snail population is evenly split between the two water points but access to water sources is heterogeneous, with 75% of contacts being made at one site (say site 1), and $R_{0}^{12}=2.5$. In this case, interventions are assumed to be able to reduce snail abundance by a fraction *ξ* of the total population. The distribution of the snail population between the two water sources does not change in case of a homogeneous intervention. With a targeted intervention, instead, the fraction of snail present at the most used water source is reduced to $\max(0,\left(n_{1}-\xi \right)/\left(1-\xi \right))$. The bottom panels of Fig. 6 show that endemic transmission could be interrupted by removing about 34% of the overall snail population with a targeted intervention, instead of 60% in the case of a blanket intervention. Unsurprisingly, then, interventions to reduce schistosomiasis transmission are increasingly more efficient (and possibly cost-effective) if targeted to the most-at-risk groups of the human population (transmission hubs) or, to a lesser extent, to the most critical water sources (transmission hotspots; see Woolhouse, Watts, Chandiwana, 1991, Woolhouse, Etard, Dietz, Ndhlovu, Chandiwana, 1998, for similar prescriptions). Remarkably, both examples show that targeted actions can interrupt endemic transmission even if transmission hubs or hotspots cannot be treated completely.

Therefore, not only does our study affirm that accounting for heterogeneity may be of paramount importance for a correct characterization of epidemicity thresholds, infection intensity patterns, and other quantities of chief epidemiological interest; it does also suggest that control measures designed under the assumption of homogeneity – of hosts’ population abundances, transmission rates and/or mobility patterns – could be misleading, and that evaluating control strategies (and their implementation) using homogeneous modeling tools could severely over- or under-estimate their effectiveness. In real-world communities, many (if not all) of the sources of heterogeneity explored in this work are likely to be in play – possibly simultaneously. These considerations raise a crucial question about how the different types of heterogeneity can be quantified from field data. To answer this question, we note that proxies of transmission risk heterogeneity can be estimated via census data, which provide details on the demographic, social and economic structure of a community, as well as on its access to safe water and sanitation (see e.g. Mari, Bertuzzo, Righetto, Casagrandi, Gatto, Rodriguez-Iturbe, Rinaldo, 2012, Mari, Gatto, Ciddio, Dia, Sokolow, De Leo, Casagrandi, 2017). This type of information could be usefully complemented with *in situ* surveys to unveil (possibly season-dependent) water-related habits and behaviors (see e.g. Woolhouse, Watts, Chandiwana, 1991, Woolhouse, Etard, Dietz, Ndhlovu, Chandiwana, 1998). Water-contact patterns can be assessed via interviews on the use of water sources, which in turn require a detailed mapping of water access points. The spatiotemporal distribution of the snail population in different water bodies can be evaluated via malacogical surveys and habitat suitability studies based on remote sensing and geospatial analysis (e.g. Brooker, 2007, Simoonga, Utzinger, Brooker, Vounatsou, Appleton, Stensgaard, Olsen, Kristensen, 2009, Stensgaard, Utzinger, Vounatsou, Hürlimann, Schur, Saarnak, Simoonga, Mubita, Kabatereine, Tchuem Tchuenté, Rahbek, Kristensen, 2013, Pedersen, Midzi, Mduluza, Soko, Stensgaard, Vennervald, Mukaratirwa, Kristensen, 2014, Perez-Saez, Mari, Bertuzzo, Casagrandi, Sokolow, De Leo, Mande, Ceperley, Frohelich, Sou, Karambiri, Yacouba, Maiga, Gatto, Rinaldo, 2015). Human mobility patterns (daily commutes and seasonal migrations) can be inferred from *ad hoc* questionnaires or, on a larger scale, from the analysis of mobile phone data (see e.g. Wesolowski, Eagle, Tatem, Smith, Noor, Snow, Buckee, 2012, Bengtsson, Gaudart, Lu, Moore, Wetter, Sallah, Rebaudet, Piarroux, 2015, Finger, Genolet, Mari, Constantin De Magny, Manga, Rinaldo, Bertuzzo, 2016, Ciddio, Mari, Sokolow, De Leo, Gatto, Casagrandi, 2017, Mari, Gatto, Ciddio, Dia, Sokolow, De Leo, Casagrandi, 2017, for epidemiological applications). Tapping one or more of these sources of information could help identify transmission hubs and hotspots, thus paving the way for more effective intervention tactics. On a cautionary side, however, we note that targeting heterogeneity may but just one (yet possibly important) piece of the jigsaw puzzle that is schistosomiasis control, and that many other crucial factors (e.g. type(s) of intervention, timing, cost-effectiveness) must be properly taken into consideration as well (see e.g. Rollinson et al., 2013).

The modeling approach analyzed in this work is clearly not exempt from limitations. One additional source of heterogeneity that is neglected in Macdonald-like models for schistosomiasis transmission is the assumption that the average parasite burden in the human community (or in a relevant subset) is an informative measure of parasite distribution in the population. Individual variations in parasite load, which are often observed in field data, could be addressed by introducing a proper stratification of infection intensity (Gurarie, King, 2014, Gurarie, King, Wang, 2010, Gurarie, King, Yoon, Li, 2016), at the expense of a relatively more complicated modeling framework. Also, human demographic dynamics, that are typically neglected in Macdonald-like models, can bear important implications for schistosomiasis dynamics and control. Human communities in developing countries may be far from demographic equilibrium (Bongaarts, 2009), thus a static stratification of the population into age-groups may be not sufficient to fully represent the actual demographic dynamics. Given the much shorter time-scales involved, spatiotemporal variations in snail demography (e.g. Perez-Saez, Mande, Ceperley, Bertuzzo, Mari, Gatto, Rinaldo, 2016, Gurarie, King, Yoon, Alsallaq, Wang, 2017) may perhaps play an even more important role in determining transmission patterns, especially in highly seasonal environments, where fluctuations of different variables (such as snail densities and human exposure rates) may be remarkably lagged. Indeed, when coupled to a realistic description of the snail intermediate hosts’ population ecology, seasonal forcing can not only influence the likelihood of parasite establishment, but also induce a wide range of endemic dynamics, including periodic, quasi-periodic and chaotic transmission patterns (Ciddio et al., 2015). Spatial coupling mechanisms are also underrepresented in this modeling framework, which, in its present form, can accommodate neither hydrological transport of the larval forms of the parasite (Maszle, Whitehead, Johnson, Spear, 1998, Lowe, Xi, Meng, Wu, Qiu, Spear, 2005) nor snail dispersal (Clennon, King, Muchiri, Kitron, 2007, Wu, Zhang, Xu, Huang, Steinmann, Utzinger, Wang, Xu, Zheng, Zhou, 2008). These shortcomings could be overcome at the expense of a more complex modeling approach describing several layers of spatial connectivity (Gurarie, Seto, 2009, Perez-Saez, Mari, Bertuzzo, Casagrandi, Sokolow, De Leo, Mande, Ceperley, Frohelich, Sou, Karambiri, Yacouba, Maiga, Gatto, Rinaldo, 2015, Ciddio, Mari, Sokolow, De Leo, Gatto, Casagrandi, 2017), possibly also accounting for the geographical signatures of environmental fluctuations (Mari et al., 2014). We finally note that a careful consideration of the spatial scale of analysis that is relevant for the application under study is crucial to determine what sources of heterogeneity are most likely to influence disease transmission.

Complemented with one or more of these possible extensions, the simple theoretical framework provided by Macdonald-like models can indeed be turned into a powerful operative tool to gain insight into the role of heterogeneity in schistosomiasis transmission dynamics, as well to analyze real-world intervention strategies.

## Appendix A Supplementary materials

Supplementary Data S1 Supplementary Raw Research Data. This is open data under the CC BY license http://creativecommons.org/licenses/by/4.0/ Supplementary Data S1

## References

[bib0001] Anderson R.M., May R.M. (1992). Infectious Diseases of Humans: Dynamics and Control.

[bib0002] Andreasen V., Christiansen F.B. (1989). Persistence of an infectious disease in a subdivided population. Math. Biosci..

[bib0003] Bacaër N., Guernaoui S. (2006). The epidemic threshold of vector-borne diseases with seasonality. J. Math. Biol..

[bib0004] Barbour A.D. (1978). Macdonald’s model and the transmission of bilharzia. Trans. R. Soc. Tropical Med. Hyg..

[bib0005] Bengtsson L., Gaudart J., Lu X., Moore S., Wetter E., Sallah K., Rebaudet S., Piarroux R. (2015). Using mobile phone data to predict the spatial spread of cholera. Sci. Rep..

[bib0006] Bittanti S., Colaneri P. (2009). Periodic Systems, Filtering and Control.

[bib0007] Bolzoni L., Real L., De Leo G.A. (2007). Transmission heterogeneity and control strategies for infectious disease emergence. PLoS One.

[bib0008] Bongaarts J. (2009). Human population growth and the demographic transition. Philos. Trans. R. Soc. B.

[bib0009] Brooker S. (2007). Spatial epidemiology of human schistosomiasis in Africa: Risk models, transmission dynamics and control. Trans. R. Soc. Tropical Med. Hygiene.

[bib0010] Centers for Disease Control and Prevention, 2011. The burden of schistosomiasis. Available online at https://www.cdc.gov/globalhealth/ntd/diseases/schisto_burden.html. Last accessed on June 30, 2017.

[bib0011] Chandiwana S.K., Christensen N.O., Frandsen F. (1987). Seasonal patterns in the transmission of *Schistosoma haematobium, S. matthei* and *S. mansoni* in the highveld region of Zimbabwe. Acta Tropica.

[bib0012] Chandiwana S.K., Woolhouse M.E.J. (1991). Heterogeneities in water contact patterns and the epidemiology of *Schistosoma haematobium*. Parasitology.

[bib0013] Ciddio M., Mari L., Gatto M., Rinaldo A., Casagrandi R. (2015). The temporal patterns of disease severity and prevalence in schistosomiasis. Chaos.

[bib0014] Ciddio M., Mari L., Sokolow S.H., De Leo G.A., Gatto M., Casagrandi R. (2017). The spatial spread of schistosomiasis: A multidimensional network model applied to Saint-Louis region, Senegal. Advances in Water Resources in press.

[bib0015] Clennon J.A., King C.H., Muchiri E.M., Kitron U. (2007). Hydrological modelling of snail dispersal patterns in Msambweni, Kenya and potential resurgence of *Schistosoma haematobium* transmission. Parasitology.

[bib0016] Colley D.G., Bustinduy A.L., Secor W.E., King C.H. (2014). Human schistosomiasis. Lancet.

[bib0017] Diekmann O., Heesterbeek J.A.P., Metz J.A.J. (1990). On the definition and the computation of the basic reproduction ratio *R*_0_ in models for infectious diseases in heterogeneous populations. J.Math. Biol..

[bib0018] Dushoff J., Levin S.A. (1994). The effects of population heterogeneity on disease invasion. Math. Biosci..

[bib0019] Feng Z., Eppert A., Milner F.A., Minchella D.J. (2004). Estimation of parameters governing the transmission dynamics of schistosomes. Appl. Math. Lett..

[bib0020] Finger F., Genolet T., Mari L., Constantin De Magny G., Manga N.M., Rinaldo A., Bertuzzo E. (2016). Mobile phone data highlights the role of mass gatherings in the spreading of cholera outbreaks. Proc. Natl. Acad. Sci. USA.

[bib0021] Funk S., S. B., Bauch C.T., Eames K.T.D., Edmunds W.J., Galvanie A.P., Klepac P. (2015). Nine challenges in incorporating the dynamics of behaviour in infectious diseases models. Epidemics.

[bib0022] Grimes J.E.T., Croll D., Harrison W.E., Utzinger J., Freeman M.C., Templeton M.R. (2014). The relationship between water, sanitation and schistosomiasis: A systematic review and meta-analysis. PLoS Neglected Tropical Diseases.

[bib0023] Grimes J.E.T., Croll D., Harrison W.E., Utzinger J., Freeman M.C., Templeton M.R. (2015). The roles of water, sanitation and hygiene in reducing schistosomiasis: A review. Parasites & Vectors.

[bib0024] Gurarie D., King C.H. (2005). Heterogeneous model of schistosomiasis transmission and long-term control: The combined influence of spatial variation and age-dependent factors on optimal allocation of drug therapy. Parasitology.

[bib0025] Gurarie D., King C.H. (2014). Population biology of *Schistosoma* mating, aggregation, and transmission breakpoints: More reliable model analysis for the end-game in communities at risk. PLoS One.

[bib0026] Gurarie D., King C.H., Wang X. (2010). A new approach to modelling schistosomiasis transmission based on stratified worm burden. Parasitology.

[bib0027] Gurarie D., King C.H., Yoon N., Alsallaq R., Wang X. (2017). Seasonal dynamics of snail populations in coastal Kenya: Model calibration and snail control. Advances in Water Resources in press.

[bib0028] Gurarie D., King C.H., Yoon N., Li E. (2016). Refined stratified-worm-burden models that incorporate specific biological features of human and snail hosts provide better estimates of *Schistosoma diagnosis*, transmission, and control. Parasites Vectors.

[bib0029] Gurarie D., Seto E.Y.W. (2009). Connectivity sustains disease transmission in environments with low potential for endemicity: Modelling schistosomiasis with hydrologic and social connectivities. J. R. Soc. Interface.

[bib0030] Heffernan J.M., Smith R.J., Wahl L.M. (2005). Perspectives on the basic reproductive ratio. J. R. Soc. Interface.

[bib0031] Hethcote H.W., Van Ark J.W. (1987). Epidemiological models for heterogeneous populations: Proportionate mixing, parameter estimation, and immunization programs. Math. Biosci..

[bib0032] Hollingsworth T.D., Pulliam J.R.C., Funk S., Truscott J.E., Isham V., Lloyd A.L. (2015). Seven challenges for modelling indirect transmission: Vector-borne diseases, macroparasites and neglected tropical diseases. Epidemics.

[bib0033] King C.H., Bertsch D. (2015). Historical perspective: Snail control to prevent schistosomiasis. PLoS Neglected Tropical Diseases.

[bib0034] Klausmeier C.A. (2008). Floquet theory: A useful tool for understanding nonequilibrium dynamics. Theor. Ecol..

[bib0035] Liang S., Maszle D., Spear R.C. (2002). A quantitative framework for a multi-group model of *Schistosomiasis japonicum* transmission dynamics and control in Sichuan, China. Acta Tropica.

[bib0036] Liang S., Seto E.Y.W., Remais J.V., Zhong B., Yang C., Hubbard A., Davis G.M., Gu X., Qiu D., Spear R.C. (2007). Environmental effects on parasitic disease transmission exemplified by schistosomiasis in western China. Proc. Natl. Acad. Sci..

[bib0037] Liang S., Spear R.C., Seto E., Hubbard A., Qiu D. (2005). A multi-group model of *Schistosoma japonicum* transmission dynamics and control: Model calibration and control prediction. Trop. Med. Int. Health.

[bib0038] Lloyd-Smith J.O., Schreiber S.J., Kopp P.E., Getz W.M. (2005). Superspreading and the effect of individual variation on disease emergence. Nature.

[bib0039] Lowe D., Xi J., Meng X., Wu Z., Qiu D., Spear R. (2005). Transport of *Schistosoma japonicum*cercariae and the feasibility of niclosamide for cercariae control. Parasitology Int..

[bib0040] Macdonald G. (1965). The dynamics of helminth infections, with special reference to schistosomes. Trans. R. Soc. Trop. Med. Hygiene.

[bib0041] Mari L., Bertuzzo E., Righetto L., Casagrandi R., Gatto M., Rodriguez-Iturbe I., Rinaldo A. (2012). Modelling cholera epidemics: The role of waterways, human mobility and sanitation. J. R. Soc. Interface.

[bib0042] Mari L., Casagrandi R., Bertuzzo E., Rinaldo A., Gatto M. (2014). Floquet theory for seasonal environmental forcing of spatially-explicit waterborne epidemics. Theor. Ecol..

[bib0043] Mari L., Gatto M., Ciddio M., Dia E.D., Sokolow S.H., De Leo G., Casagrandi R. (2017). Big-data-driven modeling unveils country-wide drivers of endemic schistosomiasis. Sci. Rep..

[bib0044] Maszle D.R., Whitehead P.G., Johnson R.C., Spear R.C. (1998). Hydrological studies of schistosomiasis transport in Sichuan Province, China. Sci. Total Environ..

[bib0045] McCreesh N., Booth M. (2013). Challenges in predicting the effects of climate change on *Schistosoma mansoni* and *Schistosoma haematobium* transmission potential. Trends Parasitology.

[bib0046] Nold A. (1980). Heterogeneity in disease-transmission modeling. Math. Biosci..

[bib0047] Pedersen U.B., Midzi N., Mduluza T., Soko W., Stensgaard A.S., Vennervald B.J., Mukaratirwa S., Kristensen T.K. (2014). Modelling spatial distribution of snails transmitting parasitic worms with importance to human and animal health and analysis of distributional changes in relation to climate. Geospatial Health.

[bib0048] Perez-Saez J., Mande T., Ceperley N., Bertuzzo E., Mari L., Gatto M., Rinaldo A. (2016). Hydrology and density feedbacks control the ecology of intermediate hosts of schistosomiasis across habitats in seasonal climates. Proc. Natl. Acad. Sci. USA.

[bib0049] Perez-Saez J., Mari L., Bertuzzo E., Casagrandi R., Sokolow S.H., De Leo G., Mande T., Ceperley N., Frohelich J.M., Sou M., Karambiri H., Yacouba H., Maiga A., Gatto M., Rinaldo A. (2015). A theoretical analysis of the geography of schistosomiasis in Burkina Faso highlights the roles of human mobility and water resources development in disease transmission. PLoS Neglected Trop. Dis..

[bib0050] Pinot de Moira A., Fulford A.J.C., Kabatereine N.B., Kazibwe F., Ouma J.H., Dunne. D.W., Booth M. (2007). Microgeographical and tribal variations in water contact and *Schistosoma mansoni* exposure within a Ugandan fishing community. Trop. Med. Int. Health.

[bib0051] Remais J., Michael E., Spear R. (2010). Modelling environmentally-mediated infectious diseases of humans: Transmission dynamics of schistosomiasis in China. Modelling Parasite Transmission and Control.

[bib0052] Rollinson, D., S., K., Levitz, S., R., S. J., Tchuem Tchuenté L. A., Garba, A., Mohammed, K. A., Schur, N., Person, B., Colley, D. G., and Utzinger, J. (2013). Time to set the agenda for schistosomiasis elimination. Acta Tropica, 128:423–440. 10.1016/j.actatropica.2012.04.013 22580511

[bib0053] Scott J.T., Diakhaté M., Vereecken K., Fall A., Diop M., Ly A., De Clercq D., de Vlas S.J., Berkvens D., Kestens L., Gryseels B. (2003). Human water contacts patterns in *Schistosoma mansoni* epidemic foci in northern Senegal change according to age, sex and place of residence, but are not related to intensity of infection. Trop. Med. Int. Health.

[bib0054] Simoonga C., Utzinger J., Brooker S., Vounatsou P., Appleton C.C., Stensgaard A.S., Olsen A., Kristensen T.K. (2009). Remote sensing, geographical information system and spatial analysis for schistosomiasis epidemiology and ecology in Africa. Parasitology.

[bib0055] Sokolow S.H., Huttinger E., Jouanard N., Hsieh M.H., Lafferty K.D., Kuris A.M., Riveau G., Senghor S., Thiam C., N’Diaye A., Sarr Faye D., De Leo G.A. (2015). Reduced transmission of human schistosomiasis after restoration of a native river prawn that preys on the snail intermediate host. Proc. Natl. Acad. Sci. USA.

[bib0056] Spear R.C., Seto E., Liang S., Birkner M., Hubbard A., Qiu D., Yang C., Zhong B., Xu F., Gu X., Davis G.M. (2004). Factors influencing the transmission of *Schistosoma japonicum* in the mountains of Sichuan province of China. Am. J. Trop. Med. Hygiene.

[bib0057] Spear R.C., Zhong B., Mao Y., Hubbard A., Birkner M., Remais J., Qiu D. (2004). Spatial and temporal variability in schistosome cercarial density detected by mouse bioassays in village irrigation ditches in Sicuan, China. Am. J. Trop. Med. Hygiene.

[bib0058] Steinmann P., Keiser J., Bos R., Tanner M., Utzinger J. (2006). Schistosomiasis and water resources development: Systematic review, meta-analysis, and estimates of people at risk. Lancet Inf. Dis..

[bib0059] Stensgaard A.S., Utzinger J., Vounatsou P., Hürlimann E., Schur N., Saarnak C.F., Simoonga C., Mubita P., Kabatereine N.B., Tchuem Tchuenté L.A., Rahbek C., Kristensen T.K. (2013). Large-scale determinants of intestinal schistosomiasis and intermediate host snail distribution across Africa: does climate matter?. Acta Tropica.

[bib0060] Sturrock R.F., Diaw O.T., Talla I., Niang M., Piau J.P., Capron A. (2001). Seasonality in the transmission of schistosomiasis and in populations of its snail intermediate hosts in and around a sugar irrigation scheme at Richard Toll, Senegal. Parasitology.

[bib0061] van den Driessche P., Watmough J. (2002). Reproduction numbers and sub-threshold endemic equilibria for compartmental models of disease transmission. Math. Biosci..

[bib0062] VanderWaal K.L., Ezenwa V.O. (2016). Heterogeneity in pathogen transmission: Mechanisms and methodology. Funct. Ecol..

[bib0063] Wesolowski A., Eagle N., Tatem A.J., Smith D.L., Noor A.M., Snow R.W., Buckee C.O. (2012). Quantifying the impact of human mobility on malaria. Science.

[bib0064] Woolhouse M.E.J., Dye C., Etard J.F., Smith T., Charlwood J.D., Garnett G.P., Hagan P., Hii J.L.K., Ndhlovu P.D., Quinnell R.J., Watts C.H., Chandiwana S.K., Anderson R.M. (1997). Heterogeneities in the transmission of infectious agents: Implications for the design of control programs. Proc. Natl. Acad. Sci. USA.

[bib0065] Woolhouse M.E.J., Etard J.F., Dietz K., Ndhlovu P.D., Chandiwana S.K. (1998). Heterogeneities in schistosome transmission dynamics and control. Parasitology.

[bib0066] Woolhouse M.E.J., Watts C.H., Chandiwana S.K. (1989). Spatial and temporal heterogeneity in the population dynamics of *Bulinus globosus* and *Biomphalaria pfeifferi*and in the epidemiology of their infection with schistosomes. Parasitology.

[bib0067] Woolhouse M.E.J., Watts C.H., Chandiwana S.K. (1991). Heterogeneities in transmission rates and the epidemiology of schistosome infection. Proc. R. Soc. London B.

[bib0068] World Health Organization, 2017. Schistosomiasis. Fact sheet n. 115. Available online at http://www.who.int/mediacentre/factsheets/fs115/en/. Last accessed on June 30, 2017.

[bib0069] Wu X.H., Zhang S.Q., Xu X.J., Huang Y.X., Steinmann P., Utzinger J., Wang T.P., Xu J., Zheng J., Zhou X.N. (2008). Effect of floods on the transmission of schistosomiasis in the Yangtze River valley, People’s Republic of China. Parasitology Int..

[bib0070] Zhang X., Gao S., Cao H. (2014). Threshold dynamics for a nonautonomous schistosomiasis model in a periodic environment. J. Appl. Math. Comput..

