# Supplementary material for: Heterogeneity in schistosomiasis transmission dynamics
Source: J Theor Biol. 2017 Nov 7;432:87–99. doi: 10.1016/j.jtbi.2017.08.015 (PMC5595357; doi:10.1016/j.jtbi.2017.08.015)
Supplement: Supplementary Data S1 — Supplementary Raw Research Data. This is open data under the CC BY license http://creativecommons.org/licenses/by/4.0/ [file mmc1.pdf]

Heterogeneity in schistosomiasis transmission dynamics

Supplementary content

Lorenzo Mari[1,†], Manuela Ciddio[1], Renato Casagrandi[1],
Javier Perez-Saez[2], Enrico Bertuzzo[2,3], Andrea Rinaldo[2,4],
Susanne H. Sokolow[5,6], Giulio A. De Leo[5],
Marino Gatto[1]

[1] Dipartimento di Elettronica, Informazione e Bioingegneria, Politecnico di Milano, 20133 Milano, Italy
[2] Laboratory of Ecohydrology, Ecole Polytechnique Fédérale de Lausanne, 1015 Lausanne, Switzerland
[3] Dipartimento di Scienze Ambientali, Informatica e Statistica,
Università Ca' Foscari Venezia, 30170 Venezia Mestre, Italy
[4] Dipartimento ICEA, Università di Padova, 35131 Padova, Italy
[5] Hopkins Marine Station, Stanford University, Pacific Grove, CA 93950, USA
[6] Marine Science Institute, University of California, Santa Barbara, CA 93106, USA
[†] Corresponding author: lorenzo.mari@polimi.it

Contents

Appendix A Stability analysis of the equilibria of Macdonald model **2**

Appendix B Stability analysis of the DFE of the multi-group/multi-source model **2**

Appendix C The multi-group/single-source case **4**

Appendix D The single-group/multi-source case **8**

Appendix E The spatially explicit case **12**

Appendix F The periodically forced case **14**

Appendix A Stability analysis of the equilibria of Macdonald model

The asymptotic stability of the disease-free equilibrium (DFE) of Macdonald's (1965) model can be studied by looking at the eigenvalues of the Jacobian matrix

$$\mathbf{J_0} = \begin{bmatrix} -\gamma & \beta N \\ \chi H & -\mu \end{bmatrix},$$

of system (1) in the main text evaluated at the DFE. Specifically, the DFE is stable if the dominant eigenvalue of $\mathbf{J_0}$ is negative (note that $\mathbf{J_0}$ is a Metzler matrix, therefore its dominant eigenvalue is real; see e.g. Horn and Johnson, 1990). A switch in the sign of the dominant eigenvalue of $\mathbf{H_0}$ from negative to positive corresponds to a switch in the sign of $\det(\mathbf{J_0}) = \gamma\mu - \beta N\chi H$ from positive to negative, because $\mathbf{J_0}$ is of even order. We can thus conclude that the DFE of model (1) in the main text is stable if $r_0 = (\beta N\chi H)/(\gamma\mu) < 1$, unstable otherwise.

Similarly, the asymptotic stability of the endemic equilibrium (EE) of model (1) in the main text can be determined based on the eigenvalues of the Jacobian matrix

$$\mathbf{J_e} = \begin{bmatrix} -\gamma & \beta N \\ \frac{\gamma\mu}{\beta N} & -\frac{\beta N\chi H}{\gamma} \end{bmatrix},$$

evaluated at the EE. Like $\mathbf{J_0}$, $\mathbf{J_e}$ is a Metzler matrix of even order, hence the EE switches from stable to unstable if $\det(\mathbf{J_e}) = \beta N\chi H - \gamma\mu$ switches from positive to negative. Therefore, the EE is positive and stable if $r_0 > 1$, unfeasible and unstable otherwhise.

The condition $r_0 = 1$ thus marks an exchange of stability between the DFE and the EE: for $r_0 < 1$ the DFE is stable, while the EE is unfeasible and unstable; at $r_0 = 1$ the two equilibria collide; and for $r_0 > 1$ the DFE is unstable, while the EE is positive and stable (transcritical bifurcation; see e.g. Kuznetsov, 1995).

Appendix B Stability analysis of the DFE of the multi-group/multi-source model

The asymptotic stability of the DFE of model (2) in the main text can be analyzed by studying the sign of the dominant eigenvalue of the $(G + S) \times (G + S)$ Jacobian matrix

$$\mathbf{J_0} = \begin{bmatrix} -\gamma\mathbf{I_G} & \beta N\mathbf{En} \\ \chi H\mathbf{C}^T\mathbf{h} & -\mu\mathbf{I_S} \end{bmatrix},$$

where $\mathbf{I_G}$ and $\mathbf{I_S}$ are the identity matrices of size G and S, respectively. The dominant eigenvalue of $\mathbf{J_0}$ is real, because $\mathbf{J_0}$ is a Metzler matrix. Also, if $\mathbf{E}$ and $\mathbf{C}$ are irreducible matrices (which is in turn equivalent to requiring that the graph associated with $\boldsymbol{\omega} = [\omega_{gs}]$ be strongly connected; see Horn and Johnson, 1990), by Perron-Frobenius theorem we can conclude that the dominant eigenvalue of $\mathbf{J_0}$ is a simple real root of the characteristic polynomial $\det(\lambda \mathbf{I_{G+S}} - \mathbf{J_0}) = 0$ (see again Horn and Johnson, 1990). A change in the stability of the DFE is thus marked by a switch of the dominant eigenvalue of $\mathbf{J_0}$ from negative to positive. As the dominant eigenvalue of $\mathbf{J_0}$ changes sign, the determinant of the matrix switches sign as well. By noting the block structure of the Jacobian

$$\mathbf{J_0} = \begin{bmatrix} \boldsymbol{\mathcal{A}} & \boldsymbol{\mathcal{B}} \\ \boldsymbol{\mathcal{C}} & \boldsymbol{\mathcal{D}} \end{bmatrix}$$

and that matrix $\boldsymbol{\mathcal{A}} = -\gamma \mathbf{I_G}$ is invertible by construction ($\gamma > 0$), with standard matrix manipulation techniques we get

$$\det(\mathbf{J_0}) = \det(\boldsymbol{\mathcal{A}}) \det\left(\boldsymbol{\mathcal{D}} - \boldsymbol{\mathcal{C}}\boldsymbol{\mathcal{A}}^{-1}\boldsymbol{\mathcal{B}}\right) .$$

Both $\boldsymbol{\mathcal{A}}$ and $\boldsymbol{\mathcal{D}}$ are scalar matrices, thus they commute with any other matrix. Therefore, the determinant of $\mathbf{J_0}$ can be written as

$$\det(\mathbf{J_0}) = (-\gamma)^G(-\mu)^S \det\left(\mathbf{I_S} - \frac{\beta N \chi H}{\gamma \mu} \mathbf{C}^T \mathbf{hEn}\right) .$$

If $G + S$ is an even [odd] number, $\det(\mathbf{J_0})$ switches from positive to negative [from negative to positive] as the dominant eigenvalue of $\mathbf{J_0}$ switches from negative to positive (and the DFE switches from stable to unstable). Either way, close to the transcritical bifurcation the instability condition for the DFE is

$$\det\left(\mathbf{I_S} - \frac{\beta N \chi H}{\gamma \mu} \mathbf{C}^T \mathbf{hEn}\right) < 0 ,$$

which is equivalent to requiring that the dominant eigenvalue $\mathcal{R}_0^{GS}$ of matrix $\mathbf{R} = r_0 \mathbf{C}^T \mathbf{hEn}$ be larger than one.

Appendix C The multi-group/single-source case

In the case of a heterogeneous human population subdivided into $G > 1$ groups, all of which share one common water source, model (2) in the main text simplifies to

$$\begin{aligned}\frac{dP_g}{dt} &= \beta\epsilon_g^E NY - \gamma P_g \\ \frac{dY}{dt} &= \chi H \sum_{g=1}^{G} h_g \epsilon_g^C P_g(1-Y) - \mu Y \ ,\end{aligned} \tag{A1}$$

where the subscript pertaining to the (unique) water source has been dropped for the sake of clarity, and $\phi_s^E = \phi_s^C = 1$ for all s's as assumed the main text. Note that the system reduces to the homogeneous case if all groups have the same transmission risk ($\epsilon_g^E = \epsilon_g^C = 1$ for all g's), or if there is a single group (i.e. if there exists a group g such that $h_g = 1$).

The DFE of model (A1) is the null column vector of size $(G+1)$. To ascertain its (in)stability properties, we observe that matrix $\mathbf{R}$ becomes a scalar in the case at hand. Therefore, the threshold parameter for stability is

$$\mathcal{R}_0^{G1} = r_0 \sum_{g=1}^{G} h_g \epsilon_g^E \epsilon_g^C \ .$$

If $\mathcal{R}_0^{G1} > 1$, the DFE of system (A1) is unstable, and the EE is characterized by strictly positive components, namely

$$\bar{P}_g^{G1} = \epsilon_g^E \frac{\beta N \chi H \sum_g h_g \epsilon_g^E \epsilon_g^C - \gamma\mu}{\gamma \chi H \sum_g h_g \epsilon_g^E \epsilon_g^C} \quad \text{and} \quad \bar{Y}^{G1} = \frac{\beta N \chi H \sum_g h_g \epsilon_g^E \epsilon_g^C - \gamma\mu}{\beta N \chi H \sum_g h_g \epsilon_g^E \epsilon_g^C} \ ,$$

and stable.

Both the threshold parameter $\mathcal{R}_0^{G1}$ and the coordinates of the EE depend on the quantity

$$U(h_g, \epsilon_g^E, \epsilon_g^C) = \sum_{g=1}^{G} h_g \epsilon_g^E \epsilon_g^C \ .$$

More specifically, the dynamics of system (A1) is heavily influenced by whether $U > 1$. To search for the extrema of this function subject to the equality constraints

$$\sum_g h_g = \sum_g h_g \epsilon_g^E = \sum_g h_g \epsilon_g^C = 1$$

one can apply the method of Lagrange multipliers (see e.g. Bertsekas, 1982) and define the Lagrangian

$$\mathcal{L} = \sum_g h_g \epsilon_g^E \epsilon_g^C + l_1 \left(\sum_g h_g - 1 \right) + l_2 \left(\sum_g h_g \epsilon_g^E - 1 \right) + l_3 \left(\sum_g h_g \epsilon_g^C - 1 \right) ,$$

where l_1, l_2 and l_3 are so-called Lagrange multipliers. By setting $\nabla \mathcal{L} = 0$, we get the following system of $3(G+1)$ equations

$$\begin{aligned}
\frac{\partial \mathcal{L}}{\partial h_g} &= \epsilon_g^E \epsilon_g^C + l_1 + l_2 \epsilon_g^E + l_3 \epsilon_g^C = 0 \\
\frac{\partial \mathcal{L}}{\partial \epsilon_g^E} &= h_g \epsilon_g^C + l_2 h_g = 0 \\
\frac{\partial \mathcal{L}}{\partial \epsilon_g^C} &= h_g \epsilon_g^E + l_3 h_g = 0 \\
\frac{\partial \mathcal{L}}{\partial l_1} &= \sum_g h_g - 1 = 0 \\
\frac{\partial \mathcal{L}}{\partial l_2} &= \sum_g h_g \epsilon_g^E - 1 = 0 \\
\frac{\partial \mathcal{L}}{\partial l_3} &= \sum_g h_g \epsilon_g^C - 1 = 0 \,,
\end{aligned}$$

the last three of which are simply the original constraints. The system admits a unique solution, namely $\epsilon_g^E = \epsilon_g^C = 1$ ($g = 1, \cdots, G$), with $l_1 = 1$ and $l_2 = l_3 = -1$, for all feasible h_g combinations (in this case, in fact, the distribution of the human host population among the different sub-groups is immaterial, since they are all characterized by the same exposure and contamination risks). This homogeneous solution yields $U = 1$.

If $\epsilon_g^E = \epsilon_g^C = \epsilon_g$, it is straightforward to verify that any feasible heterogeneous solution yields $U > 1$, i.e. that the homogeneous solution ($\epsilon_g = 1$ for all g's) represents a minimum for the constrained problem. This can also be easily seen through Jensen's inequality (Jensen, 1906), by which

$$\sum_g h_g \epsilon_g^2 \geq \left(\sum_g h_g \epsilon_g \right)^2 = 1 \,,$$

with the first inequality being strict unless all $\epsilon_g = \epsilon$, or a single $h_g = 1$. Therefore, a heterogeneous multi-group community sharing one water source is typically more prone to long-term parasite establishment than a homogeneous one, because $\mathcal{R}_0^{G1} = r_0 U \geq r_0$. Similarly, it is immediate to show that the prevalence of infected snails at the EE in a homogeneous community cannot be larger than the infected snail prevalence in a heterogeneous multi-group community sharing one water source, i.e. that $\bar{Y}^{G1} \geq \bar{Y}$. With simple algebraic manipulations, in fact, this inequality reduces again to $U \geq 1$. Along the same

lines, one could ask what human sub-groups in a heterogeneous community are characterized by a parasite burden higher than the one expected in a homogeneous community, i.e. under what conditions $\bar{P}_g^{G1} \geq \bar{P}$. This inequality reduces to

$$\epsilon_g \geq \frac{(\beta N \chi H - \gamma\mu)U}{\beta N \chi H U - \gamma\mu} = \frac{(r_0 - 1)U}{r_0 U - 1} = \epsilon^\star \,.$$

Clearly, $\epsilon^\star = 1$ in a homogeneous community (for which $U = 1$), $\epsilon^\star < 1$ otherwise. Therefore, in a heterogeneous multi-group community with one water source, even some groups whose exposure/contamination risk is below average (or, equivalently, lower than that of an equivalent homogeneous community) may have a parasite load larger than $\bar{P}$. It is also possible to prove that the average parasite burden cannot be smaller in a heterogeneous multi-group community than in a homogeneous one, i.e. that $\sum_g h_g \bar{P}_g^{G1} \geq \bar{P}_g$. With straightforward manipulations, this inequality becomes

$$\sum_g h_g \epsilon_g \geq \frac{(\beta N \chi H - \gamma\mu)U}{\beta N \chi H U - \gamma\mu} = \epsilon^\star \,.$$

By construction, $\sum_g h_g \epsilon_g = 1$, therefore the above condition in always verified in a multi-group heterogeneous community, for which $\epsilon^\star < 1$.

On the other hand, $U(h_g, \epsilon_g^E, \epsilon_g^C)$ is not always larger than one if ϵ_g^E and ϵ_g^C are left free to vary independently of each other. To show this, and to complement the results obtained with the 2-group case analyzed in the main text, here we also study scenarios in which $G > 2$ groups (for which $\epsilon_g^E \neq \epsilon_g^C$) can be distinguished within a human community. This requires the definition of a joint frequency distribution for $h_g(\epsilon_g^E, \epsilon_g^C)$, i.e. for the distribution of the human host population among classes characterized by different exposure and contamination risk.

Fig. A1A illustrates an example of a discrete bivariate distribution for community composition. The discrete distribution has been generated starting from a continuous bivariate distribution $h(\epsilon^E, \epsilon^C)$ obtained through a Gaussian copula (Nelsen, 1990) of the marginal distributions over exposure $h^E(\epsilon^E) = \int h(\epsilon^E, \epsilon^C) d\epsilon^C$ and contamination risk $h^C(\epsilon^C) = \int h(\epsilon^E, \epsilon^C) d\epsilon^E$, both assumed to be exponential. The continuous distribution $h(\epsilon^E, \epsilon^C)$ has been discretized into $G = 100$ groups, each of which is endowed with specific exposure/contamination risk. We observe (Fig. A1B), coherently with results by Woolhouse et al. (1998), that if exposure and contamination risk are uncorrelated (correlation coefficient $\rho = 0$) the endemicity thresholds of the heterogeneous and homogeneous communities coincide ($\mathcal{R}_0^{G1} = r_0 = 1$); conversely if they are positively [negatively] correlated ($\rho > 0$) [($\rho < 0$)], sub-threshold parasite endemism ($\mathcal{R}_0^{G1} > 1$ with $r_0 < 1$) [super-threshold parasite extinction ($\mathcal{R}_0^{G1} < 1$ with $r_0 > 1$)] can occur in the heterogeneous community.

Fig. A1C shows instead a multi-group community in which the marginal distributions over exposure

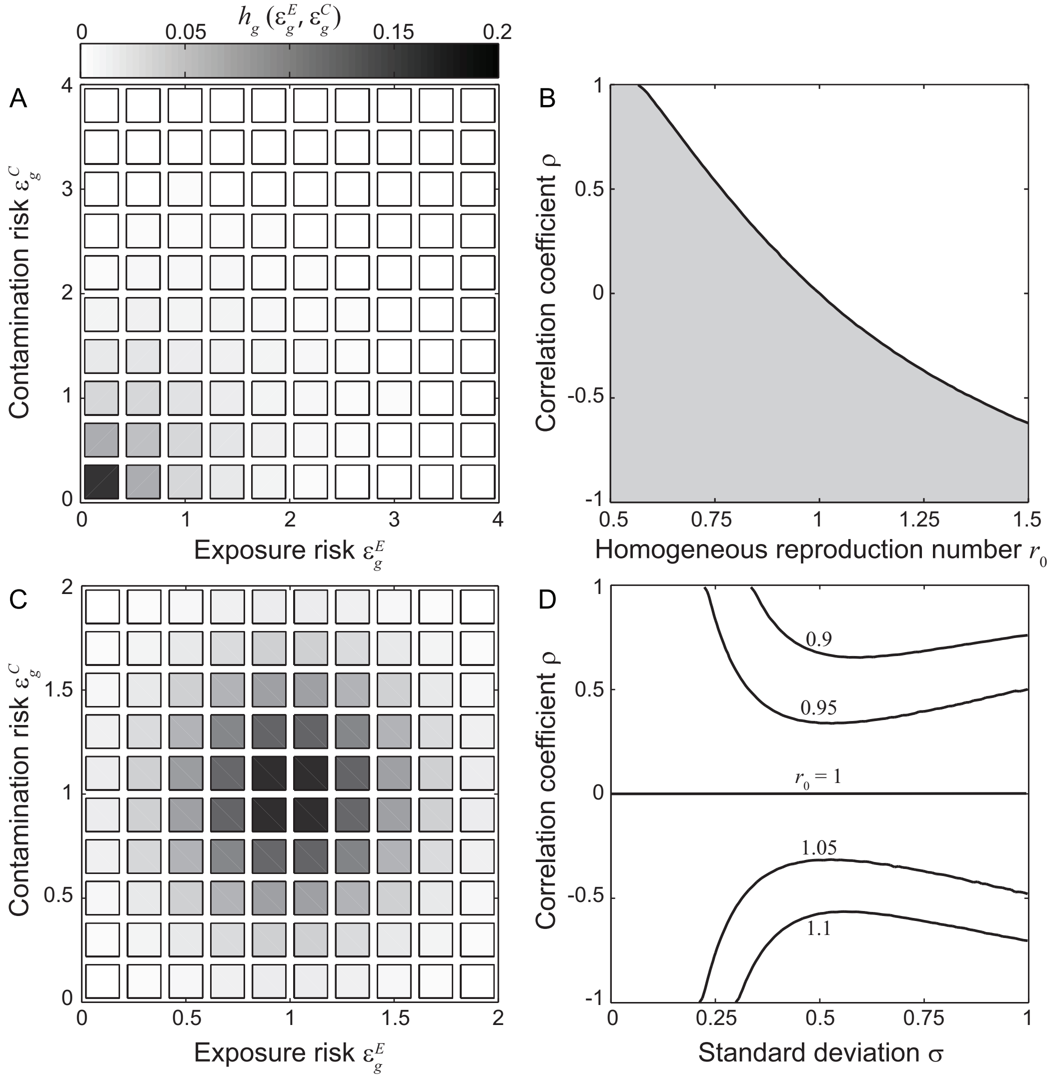

Figure A1: Analysis of multi-group communities with access to a single water source. Groups differ for their relative abundance (h_g, with $\sum_g h_g = 1$), and for their intrinsic risk of exposure and contamination (ϵ_g^E and ϵ_g^C, respectively). A) Example of subdivision of the human host population into $G = 100$ subgroups with differential exposure and contamination risk. The marginal frequency distributions over exposure ($h^E(\epsilon^E)$) and contamination ($h^C(\epsilon^C)$) risk are assumed to be exponential with mean equal to 1, while the joint frequency distribution ($h(\epsilon^E, \epsilon^C)$) is obtained through a Gaussian copula with a correlation coefficient $\rho = 0.5$. B) Endemicity boundary in the heterogeneous community (black line, $\mathcal{R}_0^{G1} = 1$). The DFE is stable if $\mathcal{R}_0^{G1} < 1$ (gray-shaded parameter combinations), while the EE is feasible and stable if $\mathcal{R}_0^{G1} > 1$. C) As in panel A, for a multi-group community in which the marginal frequency distributions over exposure and contamination risk are Gaussian with mean equal to 1 and standard deviation $\sigma = 0.4$; the joint frequency distribution is again obtained through a Gaussian copula ($\rho = 0$ in this example). D) Endemicity boundaries in the heterogeneous community. Parasite establishment is possible ($\mathcal{R}_0^{G1} > 1$) above the bifurcation curves, which correspond to $\mathcal{R}_0^{G1} = 1$ and are obtained for different values of the basic reproduction number in an equivalent homogeneous community (labels).

and contamination risk are assumed to be Gaussian (with the joint frequency distribution for $h(\epsilon^E, \epsilon^C)$ being obtained, again, through a Gaussian copula). Similarly to the previous example, the higher the correlation between exposure and contamination risk, the higher the $\mathcal{R}_0^{G1}$-to-r_0 ratio – hence the higher the likelihood of parasite endemicity. In this case, it is also possible to explore the effect of the dispersion of the two marginal distributions around their mean, as measured by their standard deviation σ (assumed to be the same for the two distributions). Interestingly, in the presence of positive [negative] correlation, sub-threshold endemic transmission [super-threshold parasite extinction] in a heterogeneous community can be found for intermediate values of σ. Because a (possibly strong) positive correlation between exposure and contamination risk is to be expected for a disease like schistosomiasis (see main text), we can conclude that the establishment of endemic pathogen transmission can be remarkably favored in heterogeneous multi-group communities.

Appendix D The single-group/multi-source case

In the case of a well-mixed human population with access to $S > 1$ common water sources, model (2) in the main text (with $\phi_s^E = \phi_s^C = 1$ for all s's) becomes

$$\begin{aligned} \frac{dP}{dt} &= \beta N \sum_{s=1}^{S} n_s \omega_s Y_s - \gamma P \\ \frac{dY_s}{dt} &= \chi \omega_s H P (1 - Y_s) - \mu Y_s \,. \end{aligned} \tag{A2}$$

Note that the subscript pertaining to the (unique) human group has been dropped for clarity, and that the system reduces to the homogeneous case if there exists a water point where all snails and human water contacts are concentrated (i.e. if there exists s such that $\omega_s = n_s = 1$). Besides the DFE, a null column vector of size $(1 + S)$, model (A2) also has a nontrivial EE solution. In this case, however, a compact analytic expression for the EE is not available.

To determine conditions under which the DFE loses stability, we note that in the single-group/multiple-source case the generalized reproduction matrix becomes $\mathbf{R} = r_0 \mathbf{u}^T \mathbf{un}$, which corresponds to the outer product of the column vectors $r_0 \mathbf{u}^T$ and $(\mathbf{un})^T$ (i.e. $\mathbf{R} = r_0 \mathbf{u}^T \otimes (\mathbf{un})^T$). As such, $\mathbf{R}$ is a rank-one matrix with only one non-zero eigenvalue, i.e. a matrix with a unique non-zero eigenvalue, $\mathcal{R}_0^{1S}$. The non-zero eigenvalue corresponds to the trace of the matrix, which can be readily evaluated as

$$\mathcal{R}_0^{1S} = \mathrm{tr}(\mathbf{R}) = r_0 \sum_{s=1}^{S} n_s \omega_s^2 \,.$$

If $\mathcal{R}_0^{G1} > 1$, the DFE of system (A1) is unstable, and the EE is stable and characterized by strictly

positive components, as it can be readily ascertained via numerical simulation of model (A2).

The stability of the DFE depends in this case on the quantity

$$V(n_s, \omega_s) = \sum_{s=1}^{S} n_s \omega_s^2 \,,$$

subject to the equality constraints

$$\sum_s n_s = \sum_s \omega_s = 1 \,.$$

More specifically, the stability of the DFE depends on whether $V > 1$. We note that $V = 0$ if there exists a source s such that $n_s = 0$ and $\omega_s = 1$, i.e. if all water contacts occur at a snail-free source. Conversely, $V = 1$ if there exists a source s such that $n_s = \omega_s = 1$, i.e. if all water contacts occur at the only site where snails can be found. This is indeed the case of a homogeneous community with access to a single water source, which represents a configuration for which V is maximized. In fact, if there exists a value of $0 < \omega_s < 1$ corresponding to $0 < n_s < 1$, then

$$\sum_s n_s \omega_s^2 < \sum_s n_s = 1$$

by construction. To show that there are no other extrema for the function $V(n_s, \omega_s)$, one can define the Lagrangian

$$\mathcal{L} = \sum_s n_s \omega_s^2 + l_1 \left(\sum_s n_s - 1 \right) + l_2 \left(\sum_s \omega_s - 1 \right) \,,$$

where l_1 and l_2 are Lagrange multipliers (see Bertsekas, 1982). By setting $\nabla \mathcal{L} = 0$, we get the following system of $2(S+1)$ equations

$$\begin{aligned}
\frac{\partial \mathcal{L}}{\partial n_s} &= \omega_s^2 + l_1 = 0 \\
\frac{\partial \mathcal{L}}{\partial \omega_s} &= 2 n_s \omega_s + l_2 = 0 \\
\frac{\partial \mathcal{L}}{\partial l_1} &= \sum_s n_s - 1 = 0 \\
\frac{\partial \mathcal{L}}{\partial l_2} &= \sum_s \omega_s - 1 = 0 \,.
\end{aligned}$$

The system admits a unique solution, namely $n_s = \omega_s = 1/S$ $(s = 1, \cdots, S)$, with $l_1 = -1/S^2$ and $l_2 = -2/S^2$, yielding $V = 1/S^2$ (≤ 1). Therefore, this unique stationary point of the Lagrangian is not an extremum for the general constrained problem.

To complement the results obtained with the 2-source case analyzed in the main text, here we also

analyze scenarios in which well-mixed human communities have access to $S > 2$ water sources. In general, the analysis of a multi-source case requires a suitable definition of the water contact matrix $\boldsymbol{\omega}$, whose structure is contingent on several nontrivial factors including, for instance, individual preferences and the spatial arrangement of the water sources, in addition to a detailed knowledge of the snail distribution pattern. We start from some prototypical cases – which were the subjects of early investigations on the topic (Barbour, 1978; Woolhouse et al., 1991) – in which snail abundance and/or water contacts are evenly distributed among the available water sources.

In the case of a uniform water contact pattern ($\omega_s = 1/S$ for all s's) and any generic feasible distribution of snail abundance, we have $\mathcal{R}_0^{1S} = r_0/S^2$. Therefore, in this case, the risk of disease endemicity ($\mathcal{R}_0^{1S} > 1$) rapidly decreases for increasing values of S (dilution effect, as noted by Woolhouse et al., 1991). In the opposite case of evenly distributed snail abundance ($n_s = 1/S$ for all s's), instead, a uniform water contact pattern ($\omega_s = 1/S$ for all s's) corresponds to a local minimum for $\mathcal{R}_0^{1S}$ compared to any other feasible distribution of water contacts, as argued by Barbour (1978). In fact, it is possible to show that the bordered Hessian

$$\mathbf{H_B} = \begin{bmatrix} 0 & 1 & 1 & \dots & 1 \\ 1 & 1 & 0 & \dots & 0 \\ 1 & 0 & 1 & \dots & 0 \\ \dots & \dots & \dots & \dots & \dots \\ 1 & 0 & 0 & \dots & 1 \end{bmatrix}$$

of the Lagrangian

$$\mathcal{L} = \frac{1}{S} \sum_s \omega_s^2 + l \left(\sum_s \omega_s - 1 \right) ,$$

where l is a Lagrange multiplier, is positive definite – which represents a sufficient condition for the stationary point of the constrained problem to be a local minimum (see Bertsekas, 1982).

Numerical simulations for these special cases are shown in Fig. A2A, where they are also contrasted against the scenario in which both snail abundance and water contacts are unevenly distributed among the available water sources. It turns out that cases in which both water contacts and snail abundance are randomly distributed are, on average, less prone to the establishment of endemic schistosomiasis transmission than cases of even snail/random contact distributions; conversely, they are more prone to endemic transmission than cases in which both water contacts and snail abundance are evenly distributed. Wide fluctuations do exist, though. Such fluctuations are well explained by the degree of correlation between the distributions of snail abundance and water contacts (Fig. A2B): not unexpectedly (see Woolhouse et al., 1998), the higher the correlation, the higher the likelihood of disease endemicity.

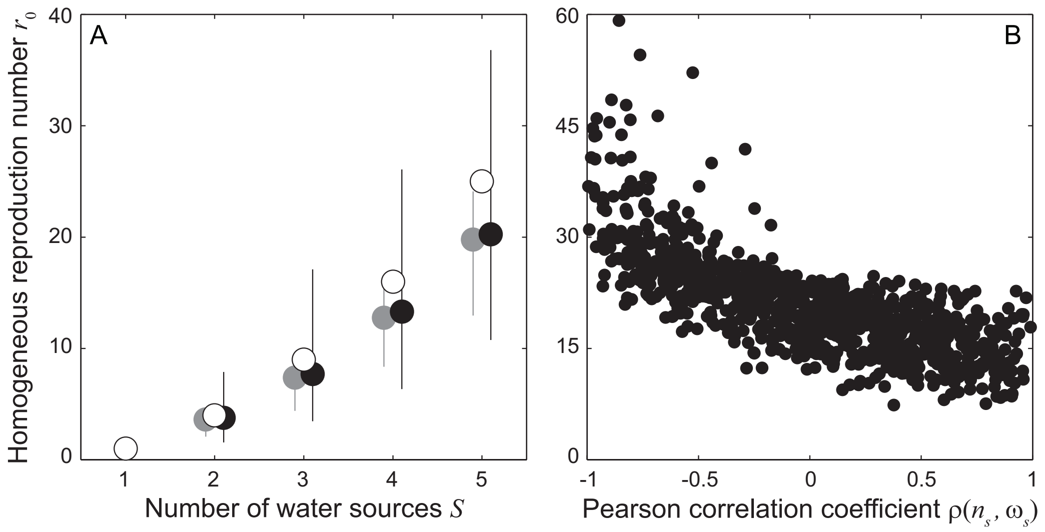

Figure A2: Analysis of single-group communities with access to multiple water sources. Sources differ for the relative abundance of snails they host (n_s, with $\sum_s n_s = 1$) and the frequency of human-water contacts (ω_s, with $\sum_s \omega_s = 1$). A) Basic reproduction number r_0 in an equivalent homogeneous community that is required for the establishment of endemic schistosomiasis transmission in a single-group community with S available water sources. White is for an even distribution of water contacts and any distribution of snail abundance, gray is for an even distribution of snail abundance and water contact patterns drawn from a uniform random distribution, black is for both distributions being assigned from a uniform random distribution; dots and vertical lines represent, respectively, average values and 95th percentile ranges evaluated over sets of 1,000 independent realizations. B) Relationship between the value of r_0 required for the establishment of endemic schistosomiasis transmission in a single-group community with $S = 5$ water sources and the correlation between the snail abundance and the water contact patterns, both of which are drawn from a uniform random distribution.

Appendix E The spatially explicit case

The simplest spatially-explicit version of model (2) in the main text can be written as

$$\begin{aligned}\frac{dP_i}{dt} &= \beta N \epsilon_i \sum_{j=1}^{2} \omega_{ij} n_j Y_j - \gamma P_i \\ \frac{dY_i}{dt} &= \chi H \sum_{j=1}^{2} \omega_{ji} \epsilon_j h_j P_j (1 - Y_i) - \mu Y_i \,,\end{aligned} \tag{A3}$$

with $i = 1, 2$. In model (A3), all the hypotheses described in the main text have been introduced, namely:

- the human population size (H) is the same as in the homogeneous case ($h_1 + h_2 = 1$),
- the snail population size (N) is the same as in the homogeneous case ($n_1 + n_2 = 1$),
- for each group, the overall human-water contact rate is the same as in the homogeneous case ($\omega_{11} + \omega_{12} = \omega_{21} + \omega_{22} = 1$),
- for each group, the intrinsic exposure risk is the same as the intrinsic contamination risk ($\epsilon_1^E = \epsilon_1^C = \epsilon_1$, $\epsilon_2^E = \epsilon_2^C = \epsilon_2$),
- the group-averaged exposure/contamination risk is the same as in the homogeneous case ($h_1\epsilon_1 + h_2\epsilon_2 = 1$),
- for each source, the intrinsic exposure risk is the same as the intrinsic contamination risk ($\phi_1^E = \phi_1^C = \phi_1$, $\phi_2^E = \phi_2^C = \phi_2$),
- the source-related exposure/contamination risk is the same across all water points ($\phi_1 = \phi_2 = 1$), and
- the fraction of water contacts made at the farthest source is the same for each groups ($\omega_{12} = \omega_{21} = m$).

Fig. 4 in the main text reports the analysis of parasite establishment conditions for four selected settings of the spatially-explicit, two-site model. Many interesting results pertain to the more complex case (panel D), in which heterogeneity is allowed in the spatial distributions of both intrinsic transmission risk and snail abundance. A similar analysis is repeated in Fig. A3 for different levels of heterogeneity in the transmission rates.

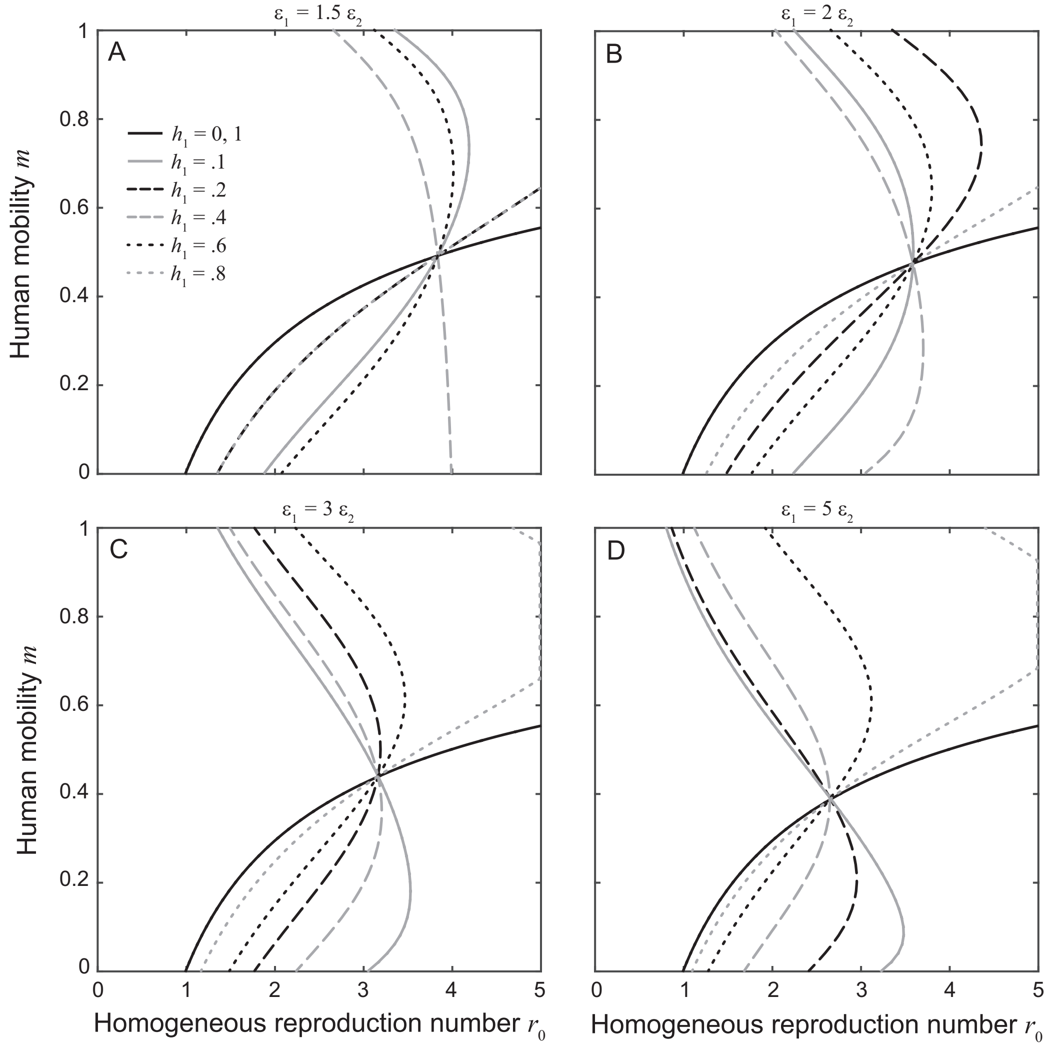

Figure A3: Endemicity boundaries in a spatially heterogeneous metacommunity with two human groups (each corresponding to a separate location) and two water sources. The groups differ for their relative abundance (h_1 and h_2, with $h_1 + h_2 = 1$) and intrinsic transmission risk (ϵ_1 and ϵ_2), while the two sources differ for the relative abundance of snails they host (n_1 and n_2, with $n_1 = h_1$ and $n_2 = h_2$) and the frequency of human-water contacts (for each community, the fraction of contacts at the farthest water point is m, while $1 - m$ is the fraction of contacts at the home site). Parasite establishment is possible ($\mathcal{R}_0^{22} > 1$) on the right of the bifurcation curves, which correspond to $\mathcal{R}_0^{22} = 1$ and are obtained for different spatial distributions of the human host population (legend). Results are shown for increasing values of transmission risk heterogeneity: A) $\epsilon_1/\epsilon_2 = 1.5$; B) $\epsilon_1/\epsilon_2 = 2$; C) $\epsilon_1/\epsilon_2 = 3$; D) $\epsilon_1/\epsilon_2 = 5$. Results obtained with $\epsilon_1/\epsilon_2 = 10$ are shown in Fig. 4D in the main text.

Appendix F The periodically forced case

After introducing all of the simplyfing hypotheses described in the main text, namely

- fluctuations of the exposure/contamination rates are associated with seasonal variations of the human-water contact rate ($\alpha_\beta = \alpha_\chi = \alpha_\omega$, $\psi_\beta = \psi_\chi = \psi_\omega$),
- fluctuations of the mortality rates of adult parasites and infected snails are neglected ($\alpha_\gamma = \alpha_\mu = 0$),
- the period of oscillations is one year for all the time-varying parameters ($\tau_\omega = \tau_N = \tau_H = \tau = 365$ [d]), and
- phase shifts between the exposure/contamination rates and the abundances of the host populations are studied with reference to the phase of the former ($\psi_\omega = 0$, $0 \leq \psi_N, \psi_H \leq \tau$),

the periodically forced model (3) in the main text becomes

$$\begin{aligned} \frac{dP}{dt} &= \tilde{\beta}(t)\tilde{N}(t)Y - \gamma P \\ \frac{dY}{dt} &= \tilde{\chi}(t)\tilde{H}(t)P(1-Y) - \mu Y \ . \end{aligned} \tag{A4}$$

In model (A4),

$$\tilde{\beta}(t) = \beta\left[1 + \alpha_\omega \sin\left(\frac{2\pi}{\tau}t\right)\right] \quad \text{and} \quad \tilde{\chi}(t) = \chi\left[1 + \alpha_\omega \sin\left(\frac{2\pi}{\tau}t\right)\right]$$

are the seasonally varying snail-to-human and human-to-snail transmission rates, while

$$\tilde{N}(t) = N\left[1 + \alpha_N \sin\left(\frac{2\pi}{\tau}(t+\psi_N)\right)\right] \quad \text{and} \quad \tilde{H}(t) = H\left[1 + \alpha_H \sin\left(\frac{2\pi}{\tau}(t+\psi_H)\right)\right]$$

are the seasonally varying population abundances of snails and humans.

References

Barbour, A. D. (1978). Macdonald's model and the transmission of bilharzia. *Transactions of the Royal Society of Tropical Medicine and Hygiene*, 72:6–15.

Bertsekas, D. P. (1982). *Constrained Optimization and Lagrange Multiplier Methods.* Academic Press, New York, USA.

Horn, R. A. and Johnson, C. R. (1990). *Matrix Analysis.* Cambridge University Press, Cambridge, USA.

Jensen, J. L. W. V. (1906). Sur les fonctions convexes et les inégalités entre les valeurs moyennes. *Acta Mathematica*, 30:175–193.

Kuznetsov, Y. A. (1995). *Elements of Applied Bifurcation Theory.* Springer-Verlag, New York, USA.

Macdonald, G. (1965). The dynamics of helminth infections, with special reference to schistosomes. *Transactions of the Royal Society of Tropical Medicine and Hygiene*, 59:489–506.

Nelsen, R. B. (1990). *An Introduction to Copulas.* Springer, New York, USA.

Woolhouse, M. E. J., Etard, J. F., Dietz, K., Ndhlovu, P. D., and Chandiwana, S. K. (1998). Heterogeneities in schistosome transmission dynamics and control. *Parasitology*, 117:475–482.

Woolhouse, M. E. J., Watts, C. H., and Chandiwana, S. K. (1991). Heterogeneities in transmission rates and the epidemiology of schistosome infection. *Proceedings of the Royal Society of London B*, 245:109–114.
